# Temporal modelling of ballast water discharge and ship-mediated invasion risk to Australia

**DOI:** 10.1098/rsos.150039

**Published:** 2015-04-22

**Authors:** Robert C. Cope, Thomas A. A. Prowse, Joshua V. Ross, Talia A. Wittmann, Phillip Cassey

**Affiliations:** 1 School of Mathematical Sciences, The University of Adelaide, Adelaide, South Australia 5005, Australia; 2 School of Biological Sciences, The University of Adelaide, Adelaide, South Australia 5005, Australia

**Keywords:** Australia, ballast water, biosecurity, invasive species, shipping, transport

## Abstract

Biological invasions have the potential to cause extensive ecological and economic damage. Maritime trade facilitates biological invasions by transferring species in ballast water, and on ships' hulls. With volumes of maritime trade increasing globally, efforts to prevent these biological invasions are of significant importance. Both the International Maritime Organization and the Australian government have developed policy seeking to reduce the risk of these invasions. In this study, we constructed models for the transfer of ballast water into Australian waters, based on historic ballast survey data. We used these models to hindcast ballast water discharge over all vessels that arrived in Australian waters between 1999 and 2012. We used models for propagule survival to compare the risk of ballast-mediated propagule transport between ecoregions. We found that total annual ballast discharge volume into Australia more than doubled over the study period, with the vast majority of ballast water discharge and propagule pressure associated with bulk carrier traffic. As such, the ecoregions suffering the greatest risk are those associated with the export of mining commodities. As global marine trade continues to increase, effective monitoring and biosecurity policy will remain necessary to combat the risk of future marine invasion events.

## 2. Introduction

Globalization of maritime trade has played a major role in facilitating the spread of species beyond their native ranges at historically unprecedented rates [1,2]. The volume of international seaborne trade has steadily increased over the past century and, at present, *ca* 90% of total world trade is carried by sea [3]. In 2012, 9.2 billion tonnes of goods were loaded in ports worldwide, compared to 8.8 billion tonnes in 2011, exceeding the same period's rate of global economic growth [4]. Assuming the current rate of growth in trade continues, as much as 23 billion tonnes of cargo could be traded annually by 2060 [5]. Seaborne trade facilitates marine species transfer between source and destination ports via two main pathways: the exchange of ballast water and hull biofouling [6].

Commercial marine vessels use ballast water to maintain trim, stability and structural integrity during voyages [7]. Ballast water is taken on or discharged both within or near ports, and during voyages. This process transports large volumes of water, together with diverse assemblages of marine organisms, across natural oceanic barriers [8]. Estimates suggest that up to 10 000 species are carried around the globe by ballast water every 24 h [9]. Ballast water discharge has been recognized as the primary invasion pathway for non-indigenous marine species transfer into coastal freshwater and marine ecosystems [10,11], although the contribution of biofouling is at least comparable to ballast water for recorded marine introductions among all vectors globally [12]. Ballast-mediated species invasions have caused extensive ecological and economic damage [13,14]. Marine invasive species have damaged economies through the fouling of infrastructure (e.g. *Dreissena polymorpha* [15]) and interactions with fisheries (e.g. *Asterias amurensis* [16]). Ecological impacts include the alteration of community structure and processes (e.g. *Mnemiopsis ledyi* [17]; *D. polymorpha* [10]; *Carcinus maenas* [18]).

Recent studies have identified high-risk, ballast-mediated invasion pathways using several methods. For example, Drake & Lodge [11] used global shipping traffic data, combined with port-to-port travel probabilities to identify global hotspots of invasion, and Kaluza *et al.* [19] identified high invasion risk pathways between global ports as those pathways with the highest connectivity. In Alaska, the volume of ballast water discharge, combined with the magnitude of shipping arrivals, was used to characterize existing invasion hotspots [20]. Given that invasion success is unlikely to be solely dependent upon shipping intensity, combinations of shipping network dynamics, environmental matching and biogeography have also been used to identify high-risk pathways and hotspots susceptible to invasion [21,22]. Seebens *et al.* [2] and Keller *et al.* [23] found that both high shipping intensity and intermediate distances between source and destination ports create higher invasion probability. Alternative methods have involved separating ports into clusters or communities based on shipping network dynamics, such that traffic within these clusters is more common than traffic between them, and assuming that key between-cluster links may provide the most significant bioinvasion risks [24]. The overarching conclusion from these studies is that invasion risk follows heavy traffic (high-connectivity) pathways.

In response to the impact of ballast-mediated species invasions, global ballast water management guidelines were introduced by the International Maritime Organization (IMO) in 1997, with further guidelines developed in 2004, subject to ongoing ratification [25,26]. One current strategy for reducing ballast-mediated invasion risk is to reduce propagule pressure via open-ocean ballast water exchange (BWE) [27,28]. BWE involves the emptying and refilling of ballast tanks mid-ocean and is based on the assumption that coastal organisms do not generally survive in mid-ocean environments and vice versa [27,28]. Several studies in North America provide empirical evidence for the effectiveness of BWE in reducing the abundance of coastal zooplankton in discharged ballast water [29–32].

To date, no studies have integrated shipping network and specific empirical ballast discharge data, including the source of discharged ballast water, to forecast potential invasion hotspots. Previous studies (e.g. [2]) have been forced to make assumptions as to where discharged ballast originated, likely due to the global paucity of comprehensive ballast surveys [2,33]. It is challenging to predict the timing and volume of ballast discharge by individual ships, as ultimately this will depend on the circumstances relevant to the journey and the discretion of the ship's master. However, historical datasets allow relative shifts in ballast discharge volumes to be quantified through time and provide a context for predictive modelling, which is essential for the effective management of global ocean biodiversity. As ballast discharge volumes are likely to increase in lockstep with global shipping trade, the ability to highlight potential ballast-mediated invasion hotspots is key for informing targeted prevention strategies. In this study, we present a case-study analysis: (i) quantifying vessel traffic, (ii) hindcasting ballast water discharge, and (iii) ranking the associated risk of biological invasion into Australian waters. Such an analysis can offer vital information for imminent policy decisions regarding the effective management of ballast water discharge by both the Australian government and IMO [1]. The Australian Department of Agriculture is currently developing new legislation (Biosecurity Bill 2014), including legislation regarding ballast water treatment and BWE, in line with the IMO ballast water management convention of 2004 [25].

Over a 2-year period between 1999 and 2001, the Australian government recorded detailed information regarding ballast uptake, exchange and discharge for all vessels visiting Australia from international waters. This rare dataset allows for quantitative predictions to be based on real network and ballast discharge data, as opposed to models that assume network characteristics [19], and is thus considerably more informative for policy decision-makers.

In this paper, we describe the temporal trends in shipping traffic into Australia from international ports between 1999 and 2012. We use the unique ballast water dataset to identify the best predictors of ballast discharge from a suite of potentially relevant ship and port characteristics. We then integrate models of ballast discharge with historical shipping network data to hindcast temporal changes in ballast release into Australian waters over the last decade. Finally, we use these outputs to rank the risk of ballast-mediated propagule transport for all shipping routes into Australia, after accounting for existing ballast exchange protocols and propagule mortality during transit.

## 3. Material and methods

### 3.1 Data

We obtained ship voyage data and ballast water census data from the Department of Agriculture (previously the Department of Agriculture, Fisheries and Forestry). The shipping data consisted of 184 249 records of individual voyages to Australian ports made by 20 325 vessels between 1999 and early 2013. Individual voyages between source and destination ports were considered as separate data points, i.e. when the same vessel visited multiple times within 1 year, each visit contributed separately. Ancillary vessel data (e.g. vessel lengths) were also available. Overall shipping data were summarized based on changes in traffic (voyage counts) over time (1999–2012) between: (i) source and destination ecoregions, (ii) distinct routes (unique pairs of source and destination ports), and (iii) vessel types.

The ballast water census data included 109 765 records of ballast uptake, exchange and discharge from individual ballast tanks, taking place over 15 386 voyages into Australia undertaken by 3440 unique vessels between 1999 and June 2001. Both the ballast uptake and discharge ports were known, as well as the last port of call and Australian destination port for each vessel.

We classified vessels into four categories: (i) bulk carriers (bulkers), (ii) tanker ships, (iii) container vessels, and (iv) ‘other’, including passenger vessels, naval vessels, general cargo, ‘Ro–Ro’ vessels (i.e. vessels transporting cars), fishing vessels and tugs. Source and destination ports were assigned to marine ecoregions based on those described by Spalding *et al.* [34] (each ecoregion was identified by both an ecoregion name and number, listed in electronic supplementary material, tables S1 and S2). Australian ports were classified into 11 broad categories based on *purpose*, i.e. the primary makeup of traffic within each port. Ports were grouped into: (i) iron ore, (ii) coal, (iii) tankers (i.e. petroleum, liquefied natural gas, liquid fuels), (iv) other metals/minerals, (v) timber, (vi) grains, (vii) sugar, (viii) imports (for ports with significantly more import traffic than export traffic), (ix) general cargo, (x) variety (for ports trafficking a broad range of cargo with no clear dominant commodity), and (xi) none-of-the-above/unknown.

All data manipulation and analyses were conducted in the R software environment for statistical computing and graphics [35].

### 3.2 Ballast water discharge analysis

We developed predictive models for ballast water discharge to assess temporal changes in: (i) the discharge volume of ballast water arriving in Australian ports and ecoregions and (ii) ballast volume from source ports and ecoregions, across vessel types. These models were then hindcast over all available shipping data. We also examined the validity of the assumption that all ballast water originated from the last port of call and that all ballast water is subsequently discharged at the destination port.

Each vessel has a carrying capacity, referred to as deadweight tonnage (DWT). DWT is the sum of the weights of cargo, fuel, ballast, passengers/crew and provisions that the ship can carry. Note that this is not the vessel's displacement. DWT is related to the size of the vessel, i.e. larger ships will (obviously) have greater capacity. We calculated DWT for each vessel as a function of vessel length by reverse-engineering a shipping design guideline. Barrass [36] suggested that when designing a ship, the intended DWT is typically known and remaining parameters are chosen to suit the cargo and intended speed. They indicated that ship length can be determined based on a ‘cube root format’

$$L={\left(\frac{\mathrm{DWT}{\left(L/B\right)}^{2}(B/H)}{pC_{B}C_{D}}\right)}^{1/3},$$

where

— *C*_D_ is the ‘deadweight coefficient’, linking deadweight to displacement, which is dependent on the intended cargo of the ship. Typical values range from 0.6 for container ships to 0.82 for bulk carriers, up to 0.85 for tankers. Passenger vessels and Ro–Ro cargo are lower (0.3–0.4).
— *C*_B_ is the ‘block coefficient’, which relates to the length, *L*, and maximum speed, *V* , of a vessel. Obtained via *C*_B_=*a*−*bV*/*L*^1/2^ with *a*=1.23 and *b*=0.395. Typically *C*_B_ ranges between 0.7 for cargo ships and 0.825 for large tankers, based upon vessel speeds in the 14–16 knot range.
— *L*/*B* is the ratio of length to breadth. Typically in the 6–7 range for ships above 130 m.
— *B*/*H* is the breadth to draft ratio. Typically around 2–2.5 for cargo vessels (3–5 for passenger vessels).
— *p*=1.025 is the density coefficient of saltwater.

When rearranged, DWT can be calculated as a linear function of *L*^3^. Based on the parameter ranges described by Barrass [36], we chose *C*_D_=0.8, *C*_B_=0.75, *L*/*B*=6.5 and *B*/*H*=2.2.

Baseline ballast capacity was estimated as 30% of calculated DWT, following recommendations provided in [37]. The resultant ballast capacity is referred to throughout as *calculated ballast capacity* (electronic supplementary material, figure S1). The ballast dataset was curated to remove data points that were obviously in error: vessels with length greater than 345 m (as no vessels of this size entered Australia within the time period), vessels with length 0 m and vessels with ballast discharge above what could be feasibly possible relative to the size of the vessel (i.e. discharge volume greater than 120% of calculated DWT). In total, approximately 16% of vessel records in the ballast water dataset were removed due to missing or clearly erroneous data.

For tankers, container vessels and other vessels, ballast discharge volume for each journey was modelled via linear multiple regression (using the MASS package [38] in R), with a separate model constructed for each vessel type. Candidate models were constructed to predict ballast discharge for each journey, based on each combination of possible predictor variables including: (i) calculated ballast capacity, (ii) destination port purpose, (iii) vessel source and destination ports, and (iv) vessel source and destination ecoregions. Bayesian information criterion (BIC) was used to select the most appropriate candidate model for each vessel type.

Ballast discharge from bulkers was not effectively modelled via multiple regression in this way, because: (i) calculated ballast capacity and port purpose show strong correlation structure (e.g. the majority of vessels in the ballast water dataset of more than 225 m in length went to ports with primary purpose: the shipment of coal or iron ore) and (ii) the variance in ballast discharged increased with calculated ballast capacity. Further, we deemed that a single ballast discharge model for bulkers was unrealistic because these vessels may discharge a typical volume of ballast water, or discharge significantly less or more (e.g. under heavy ballast conditions). Therefore, we used Gaussian mixture models [39–43] to model ballast discharge by bulkers, using the mixtools package [44] in R.

In these mixture models, the response variable is assumed to come from one of a number of different linear models or ‘components’, rather than from a single linear model (as in standard linear regression). Specifically, each vessel with calculated ballast capacity *x*_*i*_ is assumed to be drawn from model *j* with some probability *p*_*j*_, under which predicted ballast discharge $y_{i}∼\beta_{1j}+\beta_{2j}x_{i}+N(0,\sigma_{j}^{2}).$ The number of components was chosen between possible values in the range 1–5, based on Akaike information criterion (AIC, AIC3) and minimum description length (MDL), following the analysis of Hawkins *et al.* [45], who determined that MDL was the most effective metric for discriminating models with one to two components, and AIC and AIC3 were preferred with three or more components. Repeated random-sampling cross-validation (CV) was used to verify that standard linear models with multiple predictors were not substantially more informative than mixture models, based on cross-validated *R*^2^ values. CV was also used to compare CV predicted total ballast discharge with true total ballast discharge for mixture models with the chosen number of components, and a single-component linear model, so as to determine overall model prediction error.

Using the top-ranking models for ballast discharge volume from each vessel type, we hindcast ballast discharge in Australian ports for the entire period covered by the complete shipping dataset (1999–2012). We also calculated the total hindcast ballast discharge volume for each route and ecoregion.

### 3.3 Projected propagule survival

We constructed a survival model for the density of propagules in ballast water based on voyage length, following the formulation of Cordell *et al.* [29], to identify routes and ecoregions associated with the highest risk of ballast-water-mediated invasion. Organism density within ballast water was assumed to take the form *D*(*t*)=*D*(0) e^−λ*t*^, with *t* the voyage duration in days, *D*(0) the initial organism density and λ the decay parameter. Parameters following Cordell *et al.* [29] were *D*(0)=2113.489 and λ=0.161. A sensitivity analysis was undertaken to determine the influence of the choice of decay parameter λ on the relative importance of marine ecoregions, i.e. the most important ecoregion was the ecoregion that, under a given decay parameter, was projected as the source or destination of the greatest number of propagules in total across all years. In this sensitivity analysis, λ was varied between 0.01 and 0.50. Note that, for different taxa, the range of possible decay parameters varies greatly, e.g. decay parameters have been found to range from 0.005 to 0.768 over a variety of taxa, life stages and ballast water conditions [28].

In practice, the persistence of propagules is likely to be affected by changes in environmental conditions due to ballast discharge, i.e. the decay rate λ for a particular species is dependent upon environmental conditions such as salinity levels within the ballast tank, which can be greatly affected when BWE occurs. Assuming we know these decay rates, and the point at which ballast exchange occurs, we can recalibrate the predictions of propagule survival over time. We adapted the formulation of Wonham *et al.* [28]. It was assumed that a voyage takes total time *T*, BWE occurs a proportion *p* through the journey, and the proportion of propagules remaining (not removed/killed) after BWE is *r*. Before exchange, a species has decay rate *μ*_1_ and after it has decay rate *μ*_2_. We can model final propagule density as *D*(*T*)=*D*(0)*r* e^−*μ*_1_*pT*^ e^−*μ*_2_(1−*p*)*T*^=*D*(0)*r* e^−λ*T*^ with λ=*μ*_1_*p*+*μ*_2_(1−*p*) (e.g. electronic supplementary material, figure S2). Under this model, BWE is assumed to be instantaneous, and any decay that might occur through the duration of BWE is assumed to be included in *r*. In practice, *D*(0) and *r* are simply scaling factors that influence the number of emitted propagules, but we assumed these are constant regardless of voyage, and as such they do not influence the relative importance of ports or ecoregions. In the ballast water dataset, BWE tended to occur close to the middle of the journey, i.e. *p*≈0.5 (electronic supplementary material, figure S3). Vessels were estimated to travel at 15 knots, and the length of the journey used was the (approximate) geodesic shortest path-distance over water between the source and destination ports. Geodesic shortest paths were calculated based on GSHHG shoreline data [46]: a graph connecting all ports and shoreline points that could be visited directly over water was constructed using the iGraph package in R [47], and the shortest path on this graph was calculated using inbuilt shortest path functions. This is a similar approach to the calculations performed by Kaluza *et al.* [19]. Code used to perform this calculation is available online at github.com/robert-cope/ shortestPathOverWater.

## 4. Results

### 4.1 Global shipping network into Australia

**Figure 1.**
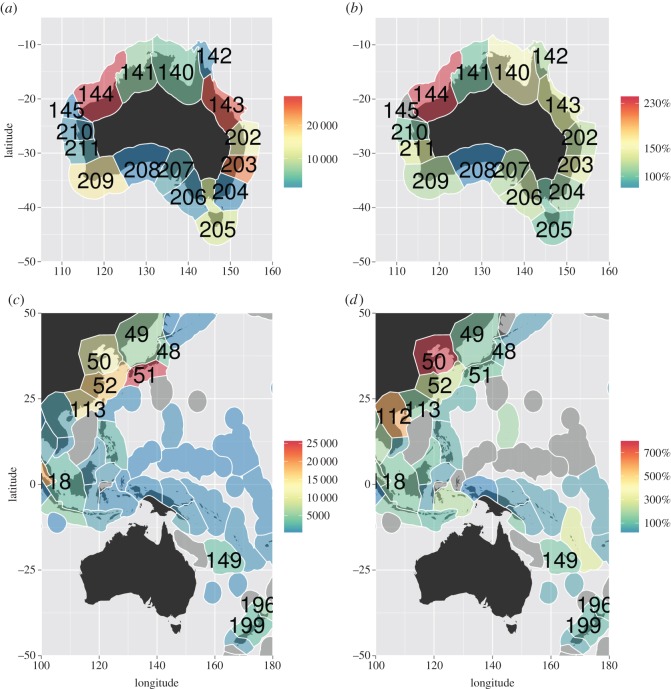
(*a*) Total number of visits to Australia, by arrival ecoregion, (*b*) proportional change in vessel traffic into Australia, by arrival ecoregion, (*c*) total number of vessels travelling to Australia, by source ecoregion, and (*d*) proportional change in vessel traffic to Australia, by source ecoregion. Grey ecoregions had no traffic. This figure includes all voyages over the full 1999–2012 time period. Numbers identify each ecoregion by code from [34]. Australian ecoregions: 140 Arnhem Coast–Gulf of Carpenteria; 141 Bonaparte Coast; 142 Torres Strait & Northern GBR; 143 Central & Southern GBR; 144 Exmouth to Broome; 145 Ningaloo; 202 Tweed–Moreton; 203 Manning–Hawkesbury; 204 Cape Howe; 205 Bassian; 206 Western Bassian; 207 South Australian Gulfs; 208 Great Australian Bight; 209 Leeuwin; 210 Shark Bay; 211 Houtman. Non-Australian ecoregions: 48 Northeastern Honshu; 49 Sea of Japan; 50 Yellow Sea; 51 Central Kuroshio Current; 52 East China Sea; 113 Southern China; 118 Malacca Strait; 149 New Caledonia; 196 Northeastern New Zealand; 199 Central New Zealand.

The three Australian ecoregions that received the most inbound shipping traffic (greatest number of journeys between 1998 and 2012; 57.6% of total voyages) were: (i) Exmouth to Broome, (ii) Central and Southern Great Barrier Reef (GBR), and (iii) Manning-Hawksbury (figure 1). Leeuwin, Tweed-Moreton and Bassian were consistently the fouth to sixth most common destinations (29% of total voyages) for inbound shipping traffic respectively (electronic supplementary material, figure S4).

Across all years in the study period, the five ecoregions from which the most shipping traffic originated (60.9% of total voyages) were, in order: (i) Central Kuroshio Current, (ii) Malacca Strait, (iii) East China Sea, (iv) Southern China, and (v) Yellow Sea (figure 1). There was some variability through time in the most prominent source ecoregions for incoming shipping traffic. From 1999 to 2010, the Central Kuroshio Current ecoregion was the most common source of shipping traffic, overtaken in 2011–2012 by the East China Sea ecoregion. From 1999 to 2005, the Malacca Strait ecoregion and the Southern China ecoregion also supplied more shipping traffic than the East China Sea ecoregion. The Yellow Sea ecoregion showed the strongest overall increase in importance over time, increasing from 11th most important source ecoregion in 1999 to fourth over the period 2008–2012 (electronic supplementary material, figure S4).

The most travelled shipping route across all years was between the ports of Singapore and Fremantle, and a number of routes outside the dominant ecoregions were among the most frequent (figure 6; electronic supplementary material, table S3). However, these individual routes tended to dominate the traffic from their corresponding ecoregions, i.e. Singapore to Fremantle accounted for more than half of the traffic between those two ecoregions and more than a quarter of total traffic into the Leeuwin ecoregion. By contrast, traffic from Central Kuroshio Current to Exmouth-Broome and Central & Southern GBR were the most frequent routes by ecoregion, but these were not dominated as strongly by one unique pair of ports, rather there were a number of ports in each ecoregion that contributed. Vessels from each of Nagoya, Yokohama and other ports from the Central Kuroshio Current ecoregion (a total of 12 of the top 50 source ports) travelled to each of Dampier, Port Hedland and Port Walcott in the Exmouth-Broome ecoregion or Gladstone, Dalrymple Bay, Hay Point, Abbott Point and others in the Central & Southern GBR ecoregion.

**Figure 2.**
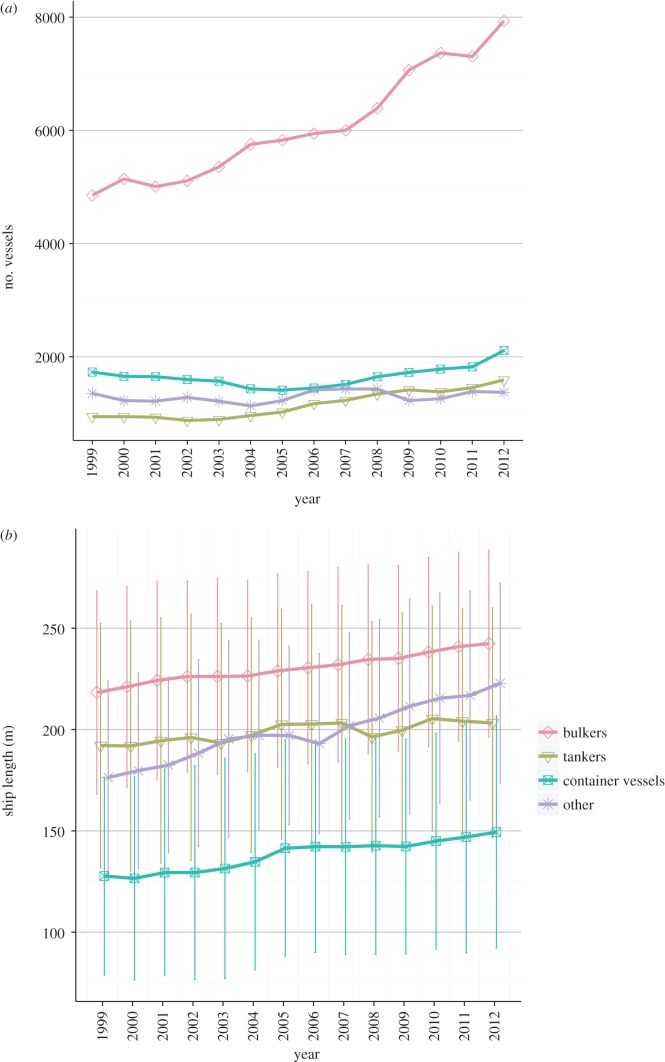
(*a*) Number of vessels arriving in Australia over time by vessel type and (*b*) distribution of lengths of vessels arriving in Australia over time by vessel type (mean ± s.d. shown).

Bulk carriers (bulkers) were both the most common vessel category, and the category that had the largest increases in traffic over the time period analysed (figure 2*a*). The ecoregions with the most overall shipping traffic (figure 1) were generally those with the most bulker traffic. Other vessel types demonstrated quite different regional traffic profiles, frequently sourced from closer ecoregions and arriving in ecoregions containing major metropolitan centres (electronic supplementary material, figures S5 and S6). While the number of bulkers arriving in Australian waters increased between 1999 and 2012, the average length of these vessels has remained relatively consistent over time. By contrast, the number of container vessels arriving has been relatively consistent, but the average size and ballast capacity of these vessels have increased through time (figure 2*b*).

### 4.2 Ballast water

In a small percentage of cases, ballast uptake and discharge locations differed from a vessel's last port of call or port of arrival, respectively, e.g. when a vessel may have taken on ballast water at a preceding port of call. Discharge location was consistent with arrival port for 92.4% of discharged ballast water (by volume). When discharge ecoregions were considered, rather than discharge ports, this increased to 98.2% of ballast water. Source ecoregions were more varied: source port and uptake port were consistent for 78.4% of ballast water by volume, increasing to 88.5% when ecoregions were considered rather than ports. When major source ecoregions were grouped, i.e. grouping ecoregions around the South China Sea (ecoregions: Gulf of Tonkin, South China Sea Oceanic Islands, Gulf of Thailand, Southern Vietnam, Sundra Shelf/Java Sea and Malacca Strait) and grouping the waters surrounding Japan and Korea (ecoregions: Northeastern Honshu, Sea of Japan, Yellow Sea, Central Kuroshio Current and East China Sea), the total proportion of ballast water for which grouped source ecoregions and grouped last-port-of-call ecoregions were consistent was 94.5%. However, there were definitely examples under which ballast water uptake occurred at a port far distant to the last port of call for a vessel, e.g. ballast water for which uptake occurred in Los Angeles, USA, but had entered Australia via Auckland, New Zealand as last port of call. Given that ballast uptake and discharge and source and destination ports were consistent in greater than three-quarters of cases, and ecoregions in *ca* 90% of cases, it was deemed acceptable to use source and destination ports as a reasonable proxy for ballast uptake and discharge locations, respectively.

For tankers, container vessels and other vessels, ballast discharge volume was modelled using multiple linear regression. In each case, the calculated ballast capacity (as a proxy for vessel size) and the assigned port purpose were significant covariates; vessel source and destination were not significant predictors. In every case, models with only port purpose and calculated ballast capacity had BIC substantially lower than models including individual ports or ecoregions (electronic supplementary material, table S5). Note that for these vessel types, there was often substantial variation between the range of vessels for which ballast data were available and those for which shipping data were available (electronic supplementary material, figure S7).

**Figure 3.**
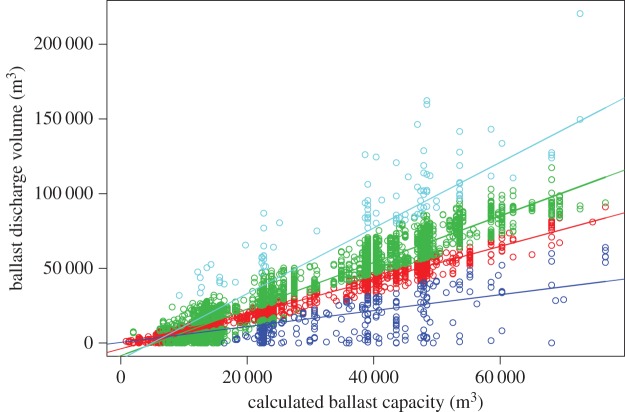
Most probable group membership for a four-component mixture model of actual ballast discharge volume versus calculated ballast capacity for bulkers. Each line indicates the linear regression (mean) for that component; coloured points correspond to the data predicted to be most likely to have arisen from that model.

Gaussian mixture models were fitted to bulker ballast data. We selected a four component model, which minimized each of AIC, AIC3 and MDL (electronic supplementary material, table S5). Repeated-measures CV was used to confirm the validity of four-component models against standard linear models, with the four-component mixture model having lower prediction error (electronic supplementary material, figure S8), lower AIC and BIC, and similar cross-validated *R*^2^ to linear models (electronic supplementary material, table S7). Predicted group memberships are shown in figure 3, model parameters in electronic supplementary material, table S8.

**Figure 4.**
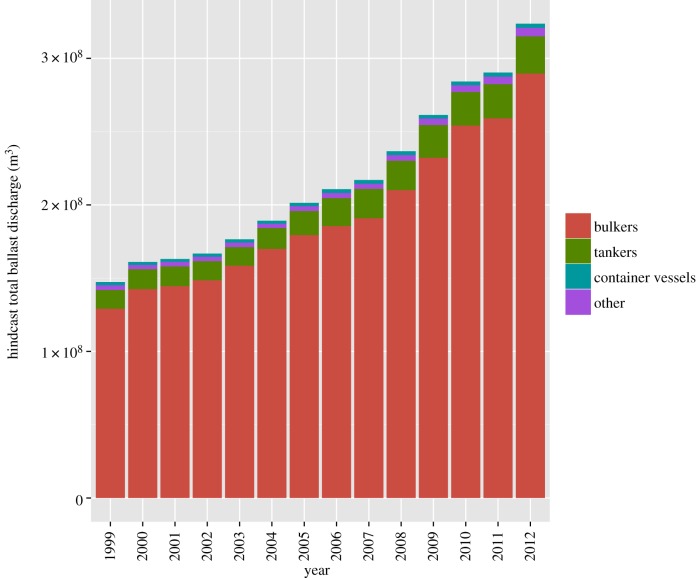
Hindcast total ballast discharge volume (*m*^3^) into Australian ports over time. Error bars were too small to be visible on this figure.

Using the linear and mixture models, we hindcast ballast water discharge data through the total shipping dataset 1999–2012. Hindcast ballast water discharge volume into Australian ports increased substantially over time, with the total hindcast ballast volume from 2012 more than double the volume discharged in 1999 (figure 4). The volume of ballast water predicted to be sourced from, or arrive at, individual ports was concentrated heavily around a small number of key ports. For example, the largest total ballast water volumes arrived at Port Hedland, Dampier and Newcastle (49.4% of total hindcast volume), and the largest volumes of ballast water were sourced from Singapore, Kaohsiung and Pohang (13.5% of total hindcast volume).

Marine ecoregions from which the most hindcast ballast water were associated tended to be those ecoregions with large amounts of bulker traffic. In particular: destination ecoregions Exmouth-Broome, Central & Southern GBR, and Manning-Hawksbury, and source ecoregions Central Kuroshio Current, East China Sea, Yellow Sea, Southern China and Malacca Strait (electronic supplementary material, figure S8).

### 4.3 Projected propagule survival

An exponential decay survival model was used to model the effect of voyage duration and BWE on the number of propagules released into Australian coastal waters via ballast water. The mean projected per-voyage density of propagules in ballast water was higher for source ecoregions closer to Australia, and into those ecoregions that received large amounts of traffic from nearby source ecoregions (electronic supplementary material, figure S10). Unsurprisingly, ecoregions with the largest volumes of ballast discharge tended to dominate total projected propagules (electronic supplementary material, figure S11).

**Figure 5.**
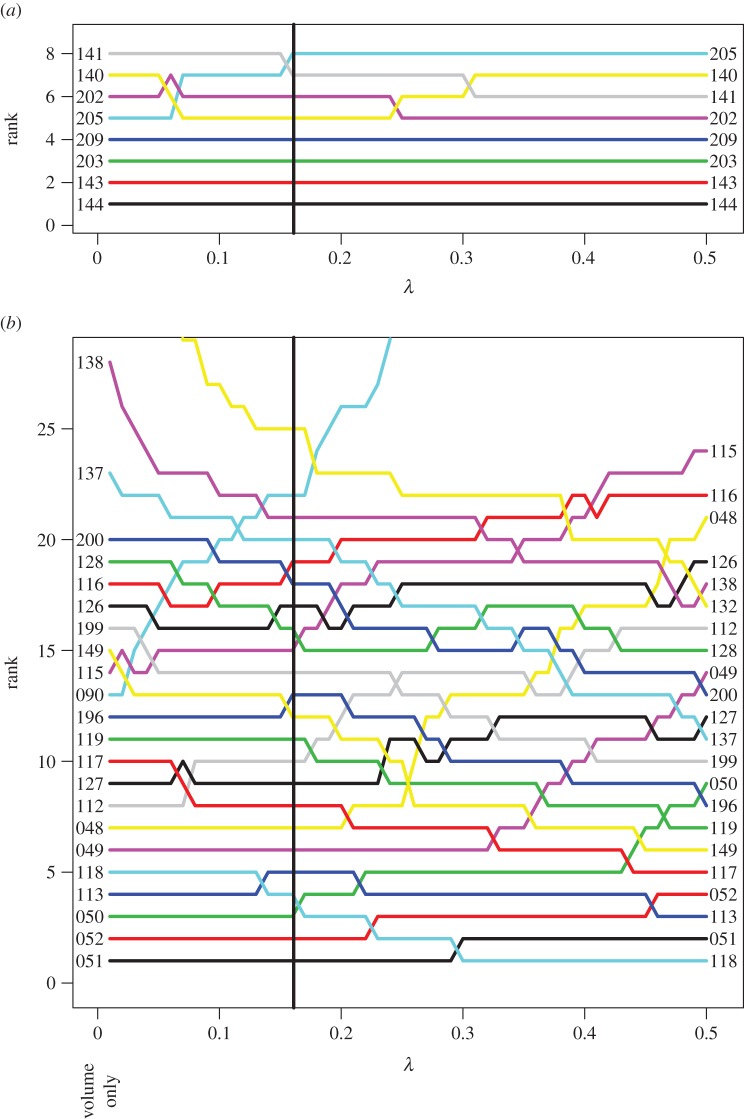
Importance of marine ecoregions based on ballast water propagules, by decay parameter λ. (*a*) Arrivals and (*b*) sources. Vertical line indicates the parameter choice from [29]. Includes all ecoregions that at any point on the scale are part of the top 8 or 17 most important ports, respectively. At the level indicated by the vertical line, these ports account for more than 95% of the projected incoming ballast propagules. Note that λ=0 implies no decay, i.e. the ranking is based solely on volume of ballast water discharged.

**Figure 6.**
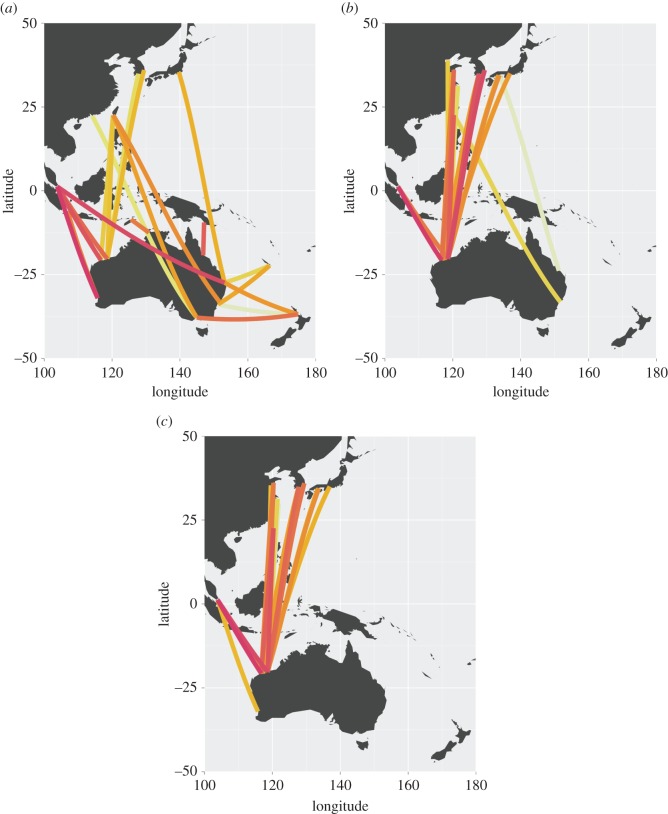
The 20 most significant routes, across all years, based on (*a*) number of voyages, (*b*) ballast water discharge volume (i.e. λ=0) and (*c*) ballast water propagules with decay at the level from [29], i.e. λ=0.16, the level indicated by the vertical lines in figure 5. Red lines indicate more important routes, decreasing in importance through orange and yellow. Lines are not indicative of actual routes nor seaborne distances between ports, only that traffic occurs between connected ports. These routes account for (*a*) 12.4%, (*b*) 16.9% and (*c*) 19.5% of voyages, total ballast water discharge and propagule pressure, respectively.

A sensitivity analysis was undertaken to investigate changes in relative risk of transport of invasive species from and to each ecoregion, varying the decay rate of propagule density in ballast water. The most important ecoregion is the one that presents the greatest risk of introduction. As the decay parameter was increased, across the range 0.01 to 0.5, closer source ecoregions increased in relative importance (figure 5). The clearest example of this is New Caledonia (149), which was ranked the 15th most important source ecoregion for ballast propagules when assuming a decay parameter of 0.01, but was the sixth most important when assuming a decay parameter of 0.5. However, source ecoregions with the largest ballast discharge volumes (particularly, the Central Kuroshio Current, East China Sea, Southern China and Malacca Strait ecoregions) remained among the most dominant source ecoregions over the full range of decay values. Similarly, the 20 most significant routes tended to be shorter when based upon propagule decay than purely on volume of ballast water discharge (figure 6). The importance of the key arrival ecoregions was consistent over the full range of parameter values.

## 5. Discussion

We characterized the spatial and temporal trends in international shipping traffic into Australia, and the associated volumes of ballast water released into Australian waters over the last decade. Shipping traffic into Australia is heavily dominated by bulk carriers (bulkers) and those ecoregions involved in bulker traffic, and these have revealed the greatest increase in frequency of visits over the period studied. Ballast water discharge was effectively modelled based on combinations of vessel type, vessel size and destination port purpose. Annual hindcast ballast water discharge volumes more than doubled over the study period (1999–2012), primarily associated with bulker traffic. These results provide a case study for ballast-water-mediated biological invasion risk, and similar techniques could be applied in other countries when relevant data are available.

### 5.1 Global shipping network into Australia

The characteristics of shipping traffic varied across different vessels types. Container ships tended to travel to ecoregions containing major metropolitan centres, whereas bulkers primarily travelled to ports near mines, which are often remote. Container and ‘other’ vessels also often arrived from relatively close regional areas including Indonesia, New Caledonia and New Zealand, whereas bulkers primarily originated from China and/or Japan.

The substantial increase in bulker traffic is correlated with economic trends in Australian export traffic over the past decade: in 2012, mining products contributed roughly 50% of total exports (by value), up from less than 25% in 1998 [48]. Further, bulker traffic is related not just to mining commodities but also to timber, grains and sugar [49]. The most notable increase in shipping traffic arriving into Australia over time was from the Yellow Sea ecoregion. Incoming traffic increased most significantly to the Exmouth-Broome and Central & Southern GBR ecoregions. These increases in traffic can be related to changes in ports within these ecoregions. For example, Port Hedland and Dampier (Exmouth-Broome ecoregion) have increased in capacity and are continuing to expand [50]. In the Central & Southern GBR ecoregion, Dalrymple Bay completed expansions in 2009 [51] (electronic supplementary material, figure S12), and Abbott Point has undergone constant expansion since 2006 [51]. This trend is likely to continue with proposed further expansions to port and/or mining capacity Australia wide.

### 5.2 Ballast water discharge

We found that total hindcast ballast water discharge into Australian waters more than doubled over the time period 1999–2012. Trends in the volume of ballast water discharge differed from overall shipping traffic patterns (i.e. the number of voyages) primarily in the sense that ballast water was discharged in greater volume from larger vessels. Those routes and ecoregions with high levels of traffic of large bulk carriers, i.e. routes and ecoregions with significant mining-related traffic, contributed more ballast water relative to those ecoregions with a lot of small vessel or non-bulker traffic.

The volume of ballast water discharged by vessels of similar size varied greatly, dependent on the type of vessel and the destination port; even when these were considered, significant variation remained. For vessels loading Australian exports, we would expect empty or near-empty vessels to enter Australia containing large volumes of ballast water to ensure vessel stability, which is discharged when vessels take on cargo while in port. However, the volume of ballast water required likely depends on the conditions at the time: the crew, weather, individual vessel nuances and any existing cargo, and these are parameters which could not feasibly be modelled in this study. As a result of this complexity, we found that a mixture model was the most effective way of modelling ballast discharge for bulkers, essentially treating the ballast water discharge model (i.e. low discharge, normal discharge or high discharge) as random for a given observation.

Mandatory ballast water treatment requirements for vessels entering Australia were introduced in mid-2001 [52], in line with existing IMO recommendations. It is possible that as a result of these changes in regulations, the ballast water discharge behaviour of vessel masters may have itself changed. Subject to new regulations, ship masters may, for example, seek to discharge smaller volumes of ballast more often when possible. However, we could find no evidence for this change in behaviour, and, as only pre-change ballast data were available, it was assumed for the purpose of this study that ballast water discharge behaviours have remained constant. This is likely to be a reasonable assumption given that ships still have to discharge significant volumes of ballast water.

Given that quantities of maritime trade are predicted to continue to increase globally into the future [3], it is likely that increases in discharged ballast water will in turn increase not just in Australia, but globally. However, it is likely that some of the trends observed here are unique to Australia. Australia is the world's top exporter of both iron ore and coking coal, driving the prevalence of bulker traffic (exporting these commodities) and associated ballast discharge. Traffic patterns, and thus ballast discharge patterns elsewhere, will depend on the economic trends in each individual country, and data in each country should be analysed in accordance with this.

### 5.3 Projected propagule survival

When a model for propagule survival based on distance is considered, and assuming mid-journey BWE, those ecoregions that are closest to Australia have on average the greatest projected propagule density (propagules per cubic metre of ballast water). However, when these outputs are projected onto hindcast ballast discharge volume data, the ecoregions with the most traffic remained of principal concern.

When the influence of the rate of decay was investigated via sensitivity analysis, the effect is unsurprising: as decay increased, closer ecoregions became increasingly more important, and distant ecoregions became less so. However, large traffic volumes remained significant: distant ecoregions which dominate traffic contribute the highest risk of propagule delivery over the full parameter range considered (figure 5).

Studies have shown that the ability of different organisms to survive in ballast water, both pre- and post-BWE, varies [28]. Different ports are likely to have different relative risk by taxa. Those taxa that survive a long time (and persist in post-BWE conditions) are likely to be more of a concern in ballast water from distant, high-traffic ports, whereas those taxa with fast decay are more likely to be of concern in ballast water from locations in closer proximity. When there is a significant difference between the decay of individuals in pre- and post- BWE conditions (e.g. due to changes in salinity or food supply), then the timing of BWE may have some influence on the overall survival of these organisms. BWE is also likely to play an important role in decreasing organism survival due to the event of BWE itself, i.e. organisms may be removed or die during the event.

### 5.4 Limitations and future directions

Until BWE or treatment options are 100% effective and thus no potentially invasive propagules survive a journey, some risk of ballast-water-mediated bioinvasion will exist [53,54]. We do not assume a known value for the effectiveness of BWE, rather noting that, as long as BWE effectiveness is constant across sources and destinations, the relative importance of each source and destination remains the same. Given the immense economic costs of biological invasions, it is necessary to continue to monitor and control potential sources of invasion, until BWE or treatment is demonstrated to be 100% effective.

Potential sources of bias throughout this study include, firstly, the assumption that ballast water discharge behaviour remains relatively similar over time. Secondly, we assume that only coastal propagules are significant and that propagules from all ecoregions are similarly important. In practice, it is possible that certain ecoregions contain more problematic potential invasive taxa, in terms of both the actual presence of taxa in the source ecoregion, and the capacity for taxa discharged from various ecoregions to survive in Australian waters when discharged (e.g. due to environmental dissimilarity). Furthermore, we assume that propagules within ballast water are relatively homogeneous in terms of both presence and survival, whereas in practice it is possible that there is some variability in the potential for propagules to survive depending on their location within a ballast tank or environmental conditions.

Transport is not the only factor that determines the success of a potential invasive species. In addition, a species must survive in its new environment, reproduce and spread [55,56]. A limitation of this study is that we do not seek to quantify invasion risk across all of these stages, but rather only the risk at the transport stage, via the relative importance of pathways and source and arrival ecoregions. Previous studies considering shipping-mediated invasion risk (e.g. [2,23]) have incorporated models for environmental similarity as a means of accounting for these other stages. We chose not to do this, as the intended focus of this study was on the transport of ballast water in particular, and incorporating further stages of the invasion process would create additional layers of complexity outside of this scope.

Future studies could involve incorporating more global shipping network data, or drawing comparisons with other countries. Further research could also investigate specifically the potentially risky taxa that could be sourced from each ecoregion of concern, or correlate recent historic ballast-water-mediated invasion events with associated shipping traffic. The impact that recent proposed ballast water treatment standards may have on the results of this study could also be considered. Further, if available, more recent ballast water data would likely improve the confidence with which predictions are made. An additional useful direction would be to develop an appropriate model capable of incorporating hull fouling, so as to consider the effects of ballast water and hull fouling on biological invasion simultaneously given that their risk profiles can be different [21]. In addition, it would be valuable to carefully compare the results of ballast water modelling with empirical field studies (e.g. [57]) detailing the presence of marine invasive species in these regions across variable levels of ballast discharge.

## 6. Conclusion

This study highlights that regardless of distance from Australia, those routes transporting larger volumes of ballast pose the greatest risk of ballast-water-mediated biological invasion. These findings will help to inform future policy targeted at the prevention and monitoring of marine bioinvasion into Australia. We recommend that targeted monitoring and management strategies focus particular attention on high-traffic ports in the Manning-Hawksbury, Central & Southern Great Barrier Reef and Exmouth to Broome regions. On a global scale, as volumes of maritime trade continues to increase, so too will the risk of ballast-water-mediated biological invasion, and constant vigilance, along with effective biosecurity management and policy, will continue to be necessary for the prevention of these marine invasion events.

## Supplementary Material

The Supplementary Material document contains all supplementary figures and tables.

## Data accessibility

Shipping and ballast water data were made available by the Department of Agriculture (formerly the Department of Agriculture, Fisheries and Forestry) under a confidentiality agreement signed on 22 July 2013. Under this agreement, results may be reported as broad-scale summary data, but no information that could be used to identify information at the level of individual ships may be disclosed. Summary figures are available in the electronic supplementary material. A table detailing the number of journeys between each pair of marine regions, by year and vessel type, is included and has been uploaded to the Dryad Digital Repository (datadyad.org;). Only those routes for which there were at least five journeys within a given year were included, so as to ensure that data pertaining to individual ships were not disclosed.

## References

[1] Hulme PE 2009 Trade, transport and trouble: managing invasive species pathways in an era of globalization. J. Appl. Ecol. 46, 10–18. (doi:10.1111/j.1365-2664.2008.01600.x)

[2] Seebens H, Gastner MT, Blasius B 2013 The risk of marine bioinvasion caused by global shipping. Ecol. Lett. 16, 782–790. (doi:10.1111/ele.12111) 23611311 10.1111/ele.12111

[3] International Maritime Organization. 2012 International shipping facts and figures. Information resources on trade, safety, security, environment. London, UK: IMO.

[4] United Nations. 2013 Review of maritime transport. Geneva, Switzerland: United Nations Conference on Trade and Development.

[5] Stopford M 2010 How shipping has changed the world and the social impact of shipping. In Global Maritime Environmental Congress, Hamburg, Germany, 7 September 2010.

[6] Drake JM, Lodge DM 2007 Hull fouling is a risk factor for intercontinental species exchange in aquatic ecosystems. Aquat. Invasions 2, 121–131. (doi:10.3391/ai.2007.2.2.7)

[7] Tsolaki E, Diamadopoulos E 2010 Technologies for ballast water treatment: a review. J. Chem. Technol. Biotechnol. 85, 19–32. (doi:10.1002/jctb.2276)

[8] Carlton JT, Geller JB 1993 Ecological roulette: the global transport of nonindigenous marine organisms. Science 261, 78–82. (doi:10.1126/science.261.5117.78) 17750551 10.1126/science.261.5117.78

[9] Carlton J 1999 The scale and ecological consequences of biological invasions in the world's oceans. In Invasive species and biodiversity management (eds Sandlund O, Schei P, Viken A), pp. 195–212. Dordrecht, The Netherlands: Kluwer.

[10] Ruiz GM, Carlton JT, Grosholz ED, Hines AH 1997 Global invasions of marine and estuarine habitats by non-indigenous species: mechanisms, extent, and consequences. Am. Zool. 37, 621–632. (doi:10.1093/icb/37.6.621)

[11] Drake JM, Lodge DM 2004 Global hot spots of biological invasions: evaluating options for ballast–water management. Proc. R. Soc. Lond. B 271, 575–580. (doi:10.1098/rspb.2003.2629) 10.1098/rspb.2003.2629 PMC1691629 15156914

[12] Hewitt C, Campbell M 2010 The relative contribution of vectors to the introduction and translocation of marine invasive species. Canberra, Australia: Australian Department of Agriculture Fisheries, and Forestry.

[13] Molnar JL, Gamboa RL, Revenga C, Spalding MD 2008 Assessing the global threat of invasive species to marine biodiversity. Front. Ecol. Environ. 6, 485–492. (doi:10.1890/070064)

[14] Frazier M, Miller AW, Ruiz GM 2013 Linking science and policy to prevent the spread of invasive species from the ballast water discharge of ships. Ecol. Appl. 23, 287–289. (doi:10.1890/11-1636.1) 23634580 10.1890/11-1636.1

[15] Pimentel D, Zuniga R, Morrison D 2005 Update on the environmental and economic costs associated with alien-invasive species in the United States. Ecol. Econ. 52, 273–288. (doi:10.1016/j.ecolecon.2004.10.002)

[16] Ross D, Johnson C, Hewitt C 2002 Impacts of an introduced seastar (*Asterias amurensis*) on a soft sediment marine assemblage: reduced recruitment of a commercial bivalve. Mar. Ecol. Prog. Ser. 241, 99–112. (doi:10.3354/meps241099)

[17] US Congress Office of Technology Assessment. 1993 Harmful non-indigenous species in the United States, OTA-F-565. US Government Printing Office, Washington, DC.

[18] Grosholz ED, Ruiz GM, Dean CA, Shirley KA, Maron JL, Connors PG 2000 The impacts of a nonindigenous marine predator in a California bay. Ecology 81, 1206–1224. (doi:10.1890/0012-9658(2000)081[1206:TIOANM]2.0.CO;2)

[19] Kaluza P, Kolzsch A, Gastner MT, Blasius B 2010 The complex network of global cargo ship movements. J. R. Soc. Interface 7, 1093–1103. (doi:10.1098/rsif.2009.0495) 20086053 10.1098/rsif.2009.0495 PMC2880080

[20] McGee S, Piorkowski R, Ruiz GM 2006 Analysis of recent vessel arrivals and ballast water discharge in Alaska: toward assessing ship-mediated invasion risk. Mar. Poll. Bull. 52, 1634–1645. (doi:10.1016/j.marpolbul.2006.06.005) 10.1016/j.marpolbul.2006.06.005 16904704

[21] Adams JK, Ellis SM, Chan FT, Bronnenhuber AG, Doolittle JE, Simard N, McKenzie CH, Martin JL, Bailey SA 2014 Relative risk assessment for ship-mediated introductions of aquatic nonindigenous species to the Atlantic Region of Canada. DFO Can. Sci. Advis. Sec. Res. Doc. 2012/116.

[22] Linley RD, Doolittle AG, Chan FT, O'Neill J, Sutherland T, Bailey SA 2014 Relative risk assessment for ship-mediated introductions of aquatic nonindigenous species to the Pacific Region of Canada. DFO Can. Sci. Advis. Sec. Res. Doc. 2013/043.

[23] Keller RP, Drake JM, Drew MB, Lodge DM 2011 Linking environmental conditions and ship movements to estimate invasive species transport accross the global shipping network. Divers. Distrib. 17, 93–102. (doi:10.1111/j.1472-4642.2010.00696.x)

[24] Xu J, Wickramarathne TL, Chawla NV, Grey EK, Steinhaeuser K, Keller RP, Drake JM, Lodge DM 2014 Improving management of aquatic invasions by integrating shipping network, ecological, and environmental data: data mining for social good. In Proc. 20th ACM SIGKDD Int. Conf. on Knowledge Discovery and Data Mining, New York, NY, 24–27 August 2014, pp. 1699–1708.

[25] International Maritime Organization. 2004 International Convention for the Control and Management of Ships' Ballast Water and Sediments.

[26] Werschkun B 2014 Emerging risks from ballast water treatment: the run-up to the International Ballast Water Management Convention. Chemosphere 112, 256–266. (doi:10.1016/j.chemosphere.2014.03.135) 25048914 10.1016/j.chemosphere.2014.03.135

[27] International Maritime Organization. 1997 Resolution A.868(20): guidelines for the control and management of ships' ballast water to minimize the transfer of harmful aquatic organisms and pathogens. London, UK: IMO.

[28] Wonham MJ, Lewis MA, MacIsaac HJ 2005 Minimizing invasion risk by reducing propagule pressure: a model for ballast-water exchange. Front. Ecol. Environ. 3, 473–478. (doi:10.1890/1540-9295(2005)003[0473:MIRBRP]2.0.CO;2)

[29] Cordell JR, Lawrence DJ, Ferm NC, Tear LM, Smith SS, Herwig RP 2009 Factors influencing densities of non-indigenous species in the ballast water of ships arriving at ports in Puget Sound, Washington, United States. Aquat. Conserv. Mar. Freshw. Ecosyst. 19, 322–343. (doi:10.1002/aqc.986)

[30] Lawrence DJ, Cordell JR 2010 Relative contributions of domestic and foreign sourced ballast water to propagule pressure in Puget Sound, Washington, USA. Biol. Conserv. 143, 700–709. (doi:10.1016/j.biocon.2009.12.008)

[31] DiBacco C, Humphrey DB, Nasmith LE, Levings CD 2011 Ballast water transport of non-indigenous zooplankton to Canadian ports. ICES J. Mar. Sci. 69, 483–491. (doi:10.1093/icesjms/fsr133)

[32] Lo VB, Levings CD, Chan KM 2012 Quantifying potential propagule pressure of aquatic invasive species from the commercial shipping industry in Canada. Mar. Poll. Bull. 64, 295–302. (doi:10.1016/j.marpolbul.2011.11.016) 10.1016/j.marpolbul.2011.11.016 22206724

[33] Ruiz GM, Hewitt C 2002 Toward understanding patterns of coastal marine invasions: a prospectus. In Invasive aquatic species of Europe. Distribution, impacts and management (eds Leppäkoski E, Gollasch S, Olenin S), pp. 529–547. Dordrecht, The Netherlands: Springer (doi:10.1007/978-94-015-9956-6_53)

[34] Spalding MD 2007 Marine ecoregions of the world: a bioregionalization of coastal and shelf areas. BioScience 57, 573–583. (doi:10.1641/B570707)

[35] R Core Team. 2014 R: a language and environment for statistical computing. Vienna, Austria: R Foundation for Statistical Computing.

[36] Barrass B 2004 Ship design and performance for masters and mates. Elsevier Science See http://books.google.com.au/books?id=2KaLDCpZgbQC.

[37] Australian Quarantine and Inspection Service. 1993 Ballast water management. Ballast Water Research Series, report no. 4 Canberra, Australia: AGPS.

[38] Venables WN, Ripley BD 2002 Modern applied statistics with S, 4th edn New York, NY: Springer See http://www.stats.ox.ac.uk/pub/MASS4.

[39] Jones PN, McLachlan GJ 1992 Fitting finite mixture models in a regression context. Aust. J. Stat. 34, 233–240. (doi:10.1111/j.1467-842X.1992.tb01356.x)

[40] McLachlan G, Peel D 2000 Finite mixture models. Wiley Series in Probability and Statistics New York, NY: John Wiley and Sons.

[41] Bohning D 2000 Computer-assisted analysis of mixtures and applications. Monographs on Statistics and Applied Probabilty, no. 81 London, UK: Chapman & Hall.

[42] Hurn M, Justel A, Robert CP 2003 Estimating mixtures of regressions. J. Comput. Graph. Stat. 12, 1–25. (doi:10.1198/1061860031347)

[43] Faria S, Soromenho G 2010 Fitting mixtures of linear regressions. J. Stat. Comput. Simul. 80, 201–225. (doi:10.1080/00949650802590261)

[44] Benaglia T, Chauveau D, Hunter DR, Young D 2009 mixtools: an R package for analyzing finite mixture models. J. Stat. Softw. 32, 1–29. (See http://www.jstatsoft.org/v32/i06/).

[45] Hawkins DS, Allend DM, Stromberg AJ 2001 Determining the number of components in mixtures of linear models. Comput. Stat. Data Anal. 38, 15–48. (doi:10.1016/S0167-9473(01)00017-2)

[46] Wessel P, Smith WHF 1996 A global self-consistent, heirachical, high-resolution shoreline database. J. Geophys. Res. 101, 8741–8743. (doi:10.1029/96JB00104)

[47] Csardi G, Nepusz T 2006 The igraph software package for complex network research. InterJ. Complex Systems, 1695. See http://igraph.org.

[48] Minifie J, Cherastidtham I, Mullerworth D, Savage J 2013 The mining boom: impacts and prospects. Melbourne, Australia: Grattan Institute.

[49] Brodie P 2015 Commercial shipping handbook, 3rd edn New York, NY: Informa Law from Routledge.

[50] Tolhurst C 2013 The expansion of our regional sea ports. Australian Financial Review. See http://www.afr.com/it-pro/the-expansion-of-our-regional-sea-ports-20130916-jh193.

[51] Lloyds. 2014 Lloyds list: Australian ports guide. See http://www.lloydslistdcn.com.au/australian_shipping_air_road_rail_services_directory/shipping-and-ports/australian-ports-guide.

[52] Department of Agriculture Fisheries and Forestry. 2013 Australian ballast water management requirements. Canberra, Australia: Australian Government.

[53] Briski E *et al* 2012 Relationship between propagule pressure and colonization pressure in invasion ecology: a test with ships' ballast. Proc. R. Soc. B 279, 2990–2997. (doi:10.1098/rspb.2011.2671) 10.1098/rspb.2011.2671 PMC3385468 22456877

[54] Briski E, Chan FT, MacIsaac HJ, Bailey SA 2014 A conceptual model of community dynamics during the tranport stage of the invasion process: a case study of ships' ballast. Divers. Distrib. 20, 236–244. (doi:10.1111/ddi.12154)

[55] Blackburn TM, Pysek P, Bacher S, Carlton JT, Duncan RP, Jarosik V, Wilson JRU, Richardson DM 2000 A proposed unified framework for biological invasions. Trends Ecol. Evol. 26, 333–339. (doi:10.1016/j.tree.2011.03.023) 21601306 10.1016/j.tree.2011.03.023

[56] Lockwood JL, Cassey P, Blackburn TM 2009 The more you introduce the more you get: the role of colonization pressure and propagule pressure in invasion ecology. Divers. Distrib. 15, 904–910. (doi:10.1111/j.1472-4642.2009.00594.x)

[57] Hewitt CL 2004 Introduced and cryptogenic species in Port Phillip Bay, Victoria, Australia. Mar. Biol. 144, 183–202. (doi:10.1007/s00227-003-1173-x)

