# Supplementary material for: Temporal modelling of ballast water discharge and ship-mediated invasion risk to Australia
Source: R Soc Open Sci. 2015 Apr 22;2(4):150039. doi: 10.1098/rsos.150039 (PMC4448877; doi:10.1098/rsos.150039)
Supplement: The Supplementary Material document contains all supplementary figures and tables. [file rsos150039supp1.pdf]

Temporal modelling of ballast water discharge and ship-mediated invasion risk to Australia: Supplementary Material

Robert C. Cope, Thomas A.A. Prowse, Joshua V. Ross, Talia A. Wittmann, and Phillip Cassey

Tables

Table S1: Correspondence between ecoregion numbers and names for Australia, from Spalding *et al.* (2007).

Ecoregion code	Ecoregion description
209	Leeuwin
211	Houtman
210	Shark Bay
145	Ningaloo
144	Exmouth to Broome
141	Bonaparte Coast
140	Arnhem Coast to Gulf of Carpenteria
142	Torres Strait Northern Great Barrier Reef
143	Central and Southern Great Barrier Reef
202	Tweed-Moreton
203	Manning-Hawkesbury
204	Cape Howe
205	Bassian
206	Western Bassian
207	South Australian Gulfs
208	Great Australian Bight

Table S2: Correspondence between ecoregion numbers and names for key ecoregions in south-east Asia, from Spalding *et al.* (2007).

Ecoregion Code	Ecoregion description
48	Northeastern Honshu
49	Sea of Japan
50	Yellow Sea
51	Central Kuroshio Current
52	East China Sea
113	Southern China
118	Malacca Strait
149	New Caledonia
196	Northeastern New Zealand
199	Central New Zealand

Table S3: Most common routes between pairs of ports for all vessels entering Australia across 1999–2012.

From	To	Count
SINGAPORE	FREMANTLE	2584
SINGAPORE	BRISBANE	1956
SINGAPORE	DAMPIER	1403
PORT MORESBY	TOWNSVILLE	949
SINGAPORE	PORT HEDLAND	898
AUCKLAND	MELBOURNE	863
DILI	DARWIN	819
JAKARTA, JAVA	FREMANTLE	750
KAOHSIUNG	NEWCASTLE	701
AUCKLAND	BRISBANE	663
KAOHSIUNG	MELBOURNE	661
NOUMEA	SYDNEY	637
YOKOHAMA	BRISBANE	609
POHANG	PORT HEDLAND	605
KAOHSIUNG	PORT HEDLAND	603
KAOHSIUNG	DAMPIER	597
NOUMEA	BRISBANE	590
KWANGYANG	PORT HEDLAND	587
HONG KONG	MELBOURNE	585
AUCKLAND	SYDNEY	561

Table S4: Most common routes between source and destination ecoregions for vessels entering Australia. Ecoregion numbers correspond to those described in supplementary Tables 1 & 2.

Source	Destination	Count
51	144	6830
51	143	6752
52	144	5325
51	203	5170
118	209	4896
50	144	4857
52	143	4498
113	203	3759
52	203	3510
118	144	3509
50	143	3161
113	144	2837
118	202	2831
113	143	2475
51	202	2248
49	144	1974
50	203	1790
113	205	1681
119	209	1585
48	203	1563

Table S5: Model selection for tankers, container vessels, and 'other' vessels. Bayesian Inference Criterion (BIC) comparisons for linear models regressed on calculated ballast capacity (CBC) and (i) port purpose, (ii) port purpose and arrival ecoregion, and (iii) port purpose and source ecoregion. The best models are those with smallest BIC.

Vessel type	CBC only	Port purpose	Port purpose and source/arrival ecoregions	Port purpose and source/arrival ports
Tankers	16098	*15965*	16133	16882
Container	12319	*12267*	12682	12523
Other	10403	*10264*	10616	10413

Table S6: Model selection table for mixture models on bulker data. Compares Akaike Information Criterion (AIC & AIC3), and Minimum Description Length (MDL) to determine an appropriate choice of k, the number of mixture components. The best models are those with the smallest values in each criteria.

k	AIC	AIC3	MDL
1	223525	223527	223539
2	220405	220415	220463
3	218519	218531	218606
4	*218028*	*218044*	*218145*
5	218212	218232	218357

Table S7: Model selection table for bulkers. Compares Akaike Information Criterion (AIC), Bayesian Information Criterion (BIC), and cross-validated (CV) R^2 values between a four-component mixture model on calculated ballast capacity (CBC), and generalized linear models regressed on CBC, CBC with source ecoregion, and CBC with port purpose. The best models are those with smallest AIC and BIC.

Model	AIC	BIC	CV R^2
4-component mixture	*218028*	*218145*	0.835
Discharge $\sim$ CBC	223525	223539	0.835
Discharge $\sim$ CBC*[source ecoregion]	222938	223054	0.844
Discharge $\sim$ CBC*[destination port purpose]	223224	223369	0.840

Table S8: Fitted parameters for a four-component mixture model for bulker data. Ballast discharge is regressed on calculated ballast capacity.

	Component 1	Component 2	Component 3	Component 4
proportion	0.43	0.50	0.05	0.02
β_1	-3932.41	-8364.72	485.55	-12123.76
β_2	1.15	1.56	0.53	2.23
σ	3433.73	6886.25	10268.12	20622.63

Figures

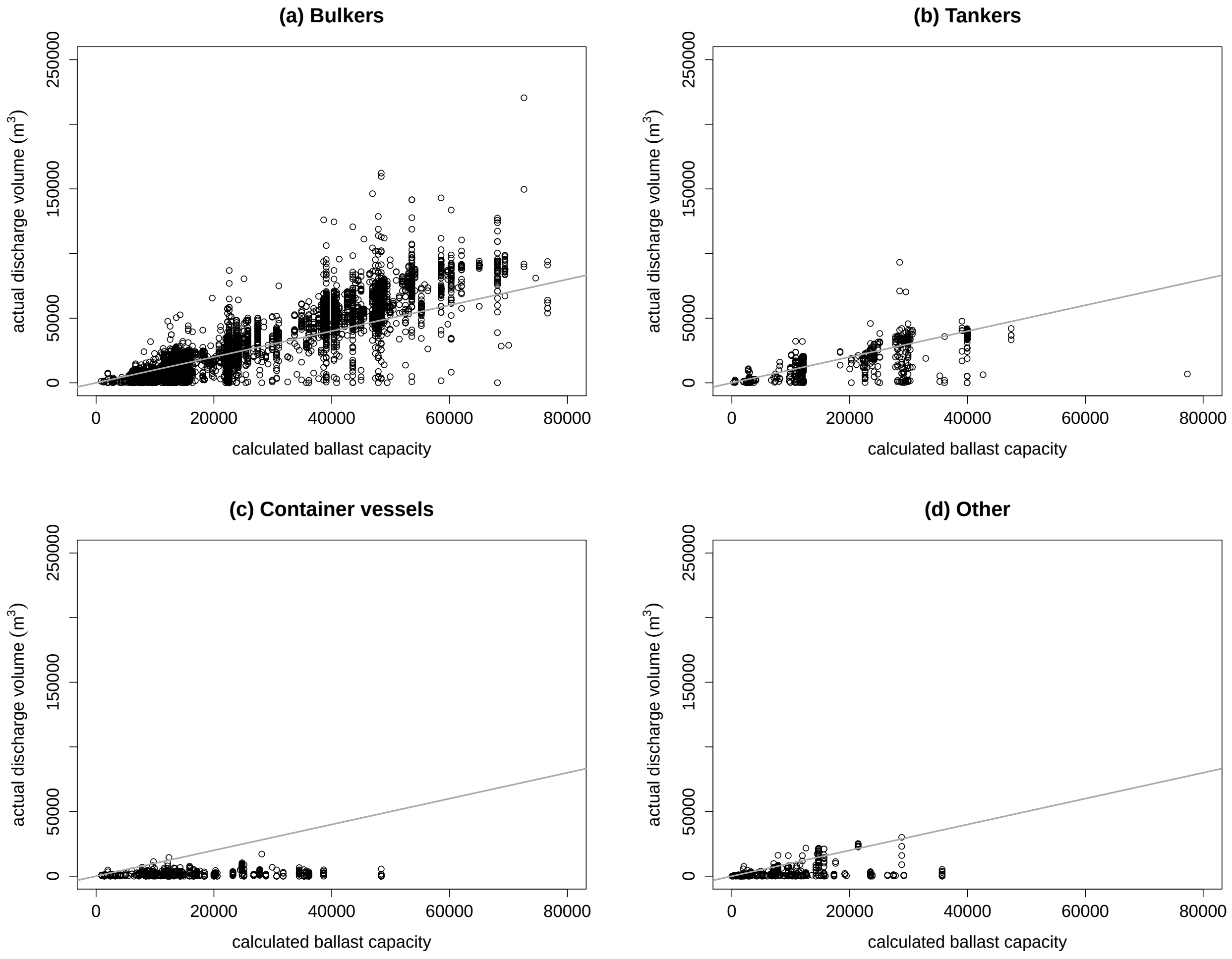

Figure S1: Actual ballast discharge vs. calculated ballast capacity for vessels of different types. The grey line indicates $y = x$, i.e, points on this line would show perfect correspondence between actual discharge and calculated ballast capacity.

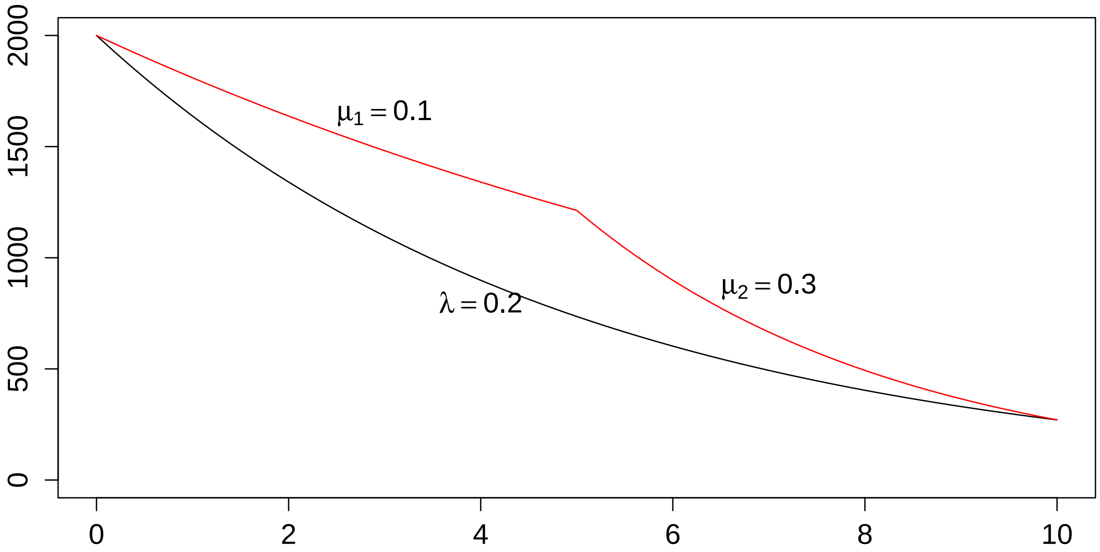

Figure S2: Example of the relationship between decay rates before and after a BWE event (i.e., μ_1 and μ_2), and the aggregate decay rate λ. In this example, BWE occurs half way though the journey ($p = 0.5$), with parameters $D(0) = 2000,\ r = 1$.

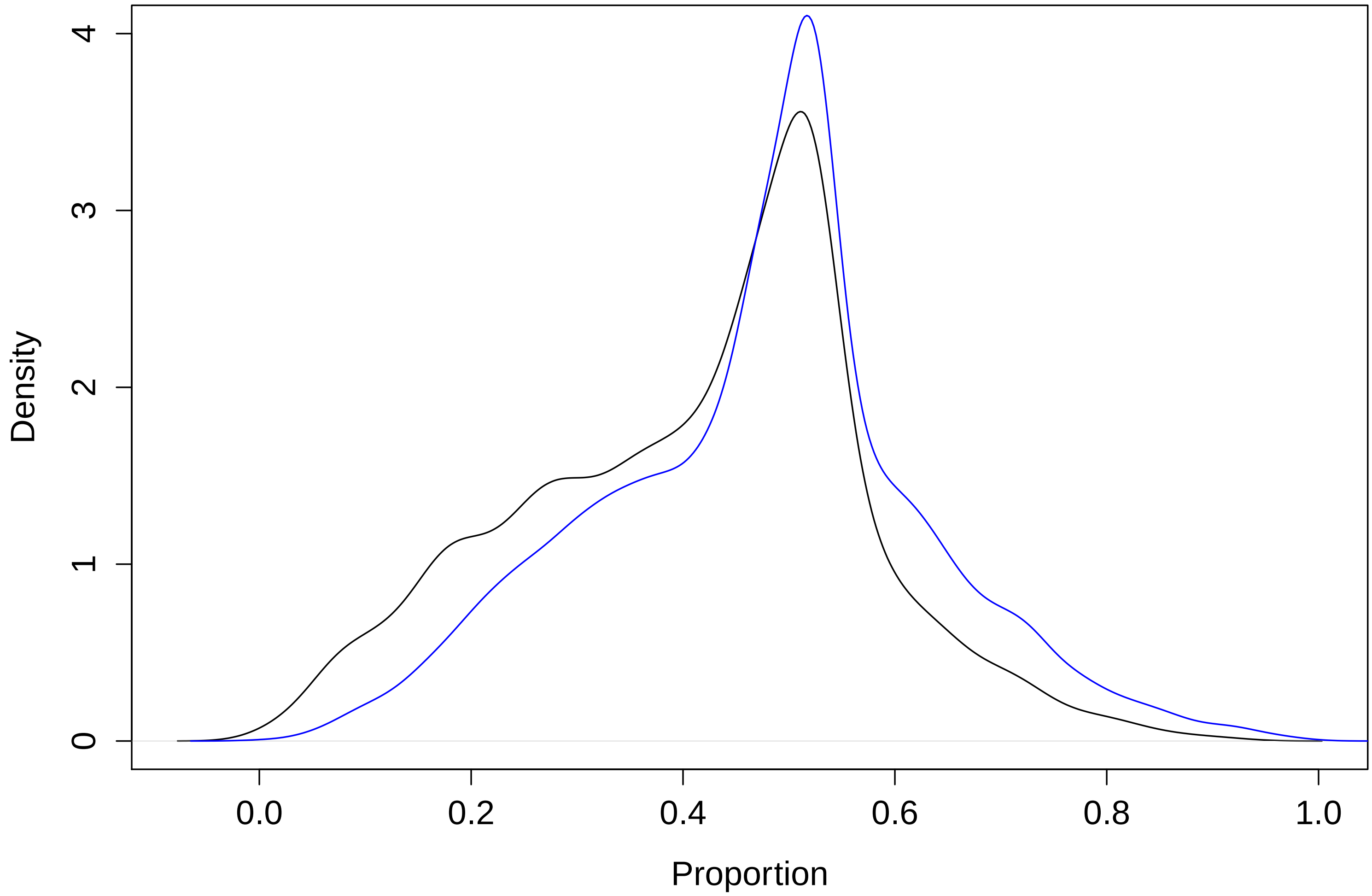

Figure S3: Density plot of the proportion of the distance through each journey at which BWE began (black) and ended (blue), across all vessels in the ballast water dataset.

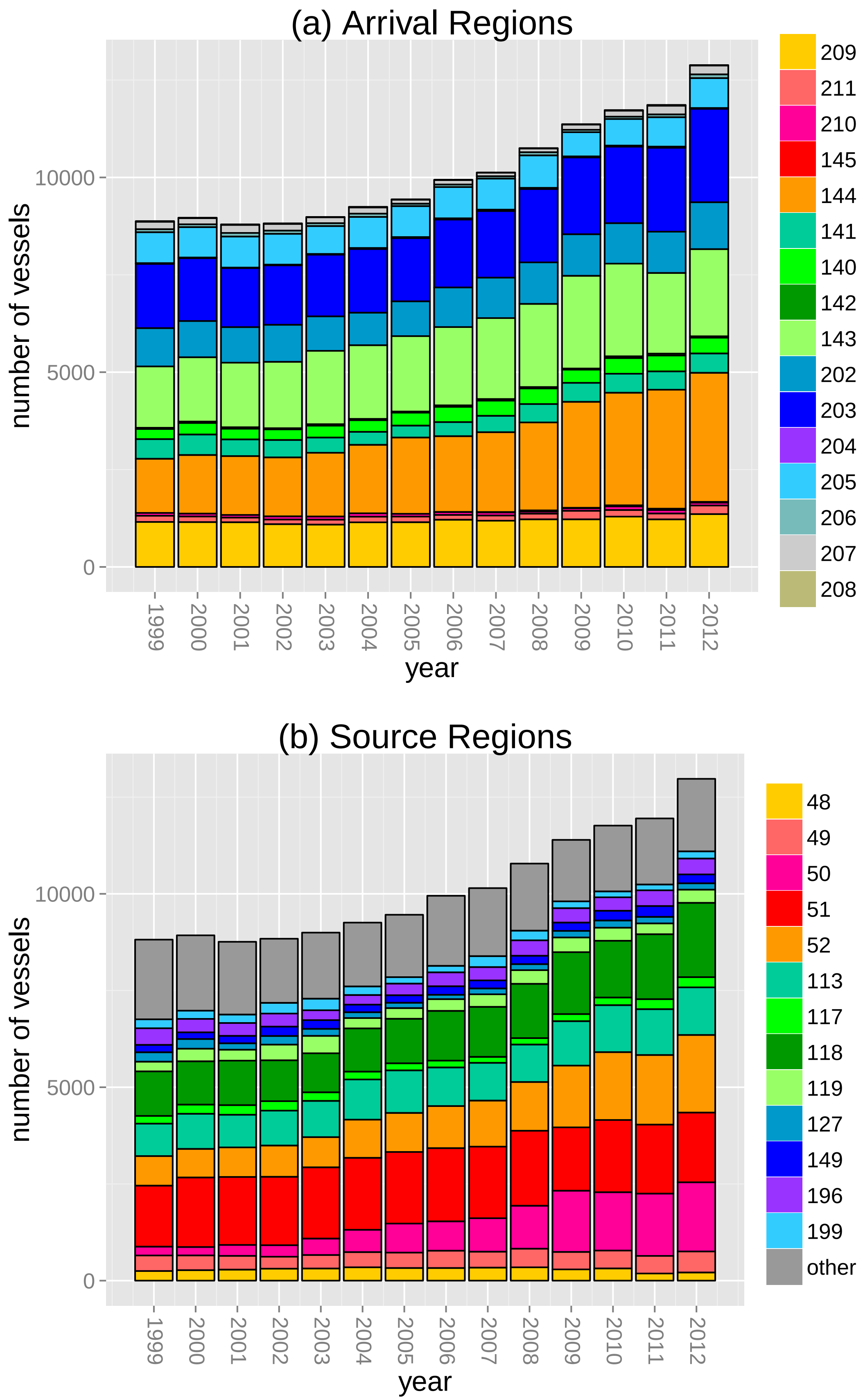

Figure S4: Ship traffic (i.e., number of vessels arriving or departing) by ecoregion over time: (a) destination ecoregions, (b) source ecoregions. Ecoregion labels correspond to those in supplementary Tables 1, 2.

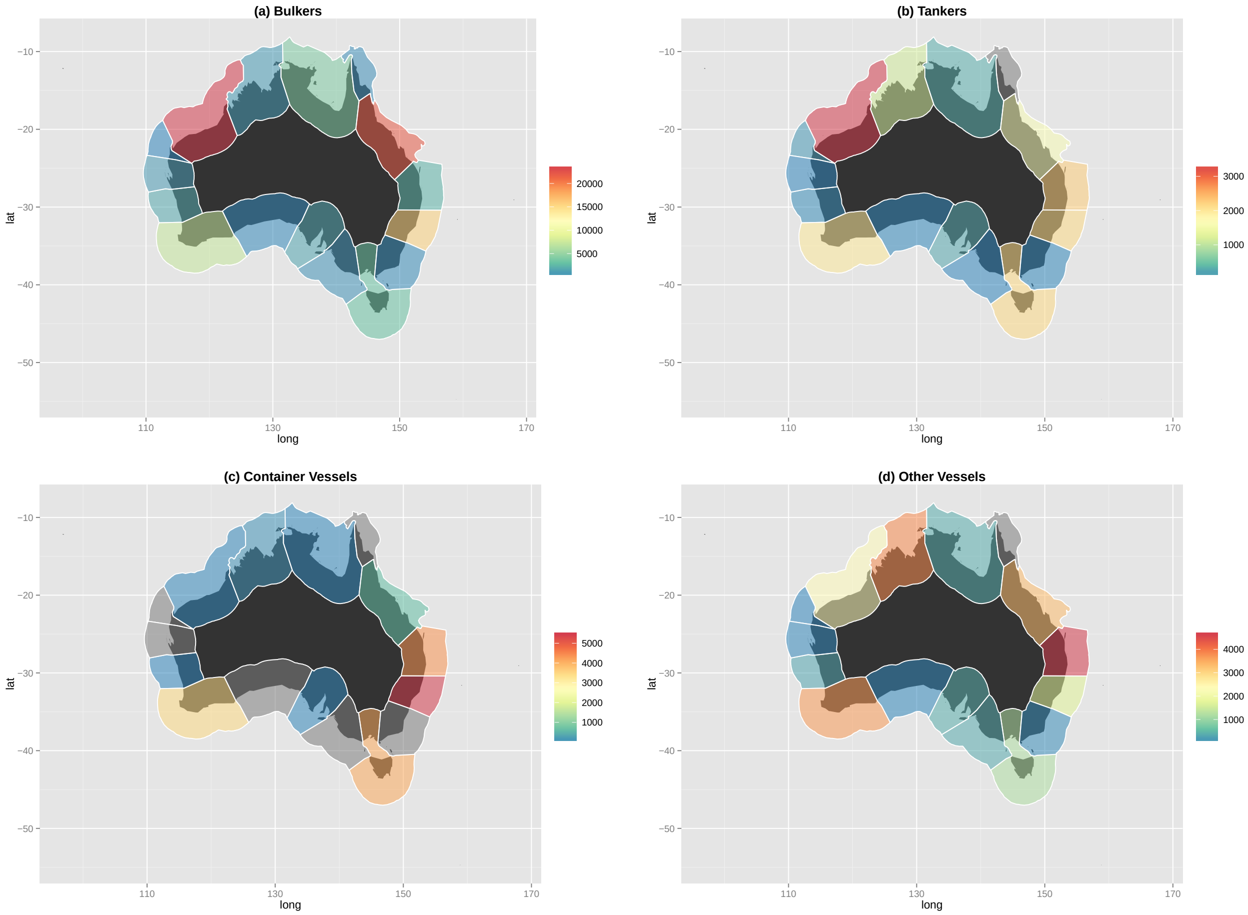

Figure S5: Total number of visits to each ecoregion, by ship type: (a) Bulkers, (b) Tankers, (c) container vessels, (d) other.

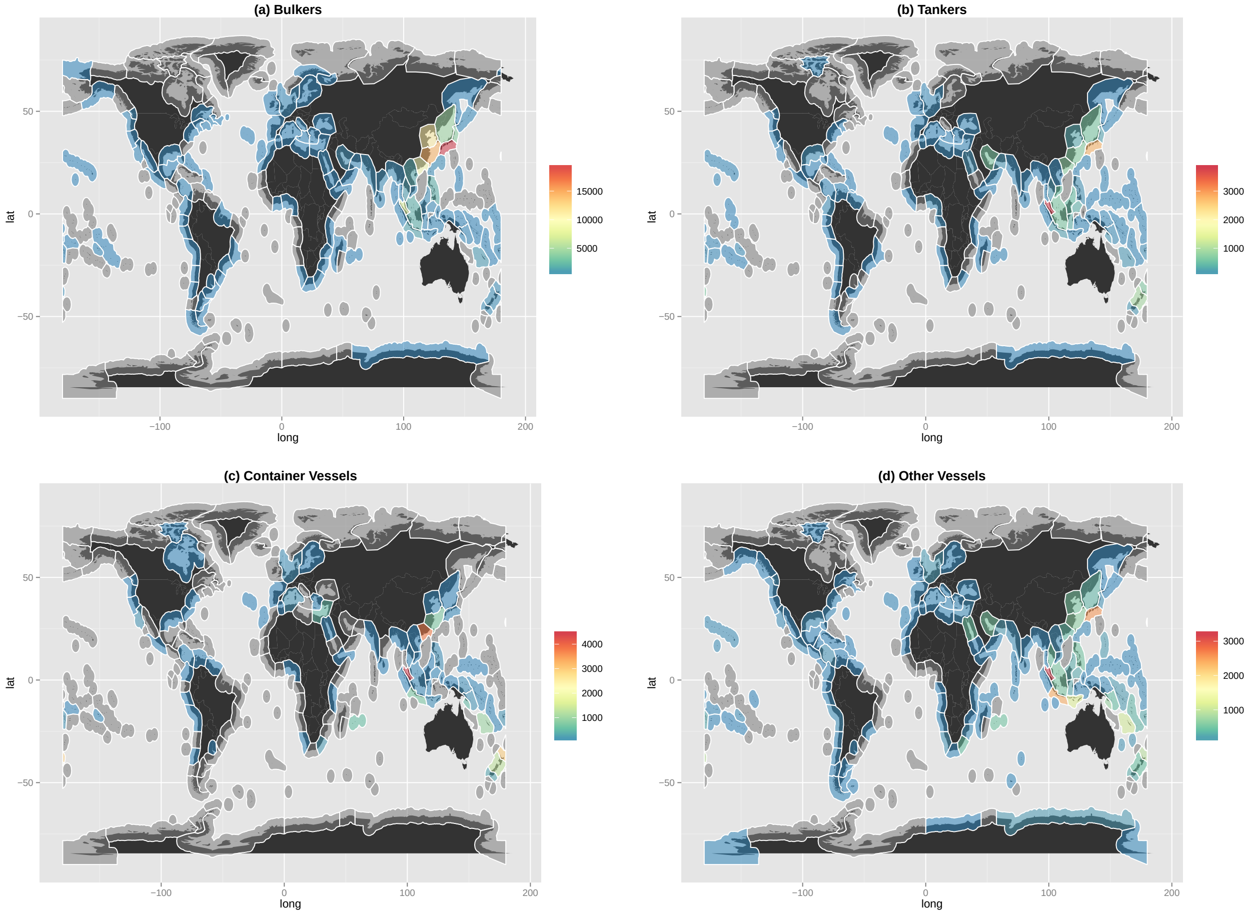

Figure S6: Total number of voyages from each ecoregion (travelling to Australia) by ship type: (a) Bulkers, (b) Tankers, (c) container vessels, (d) other.

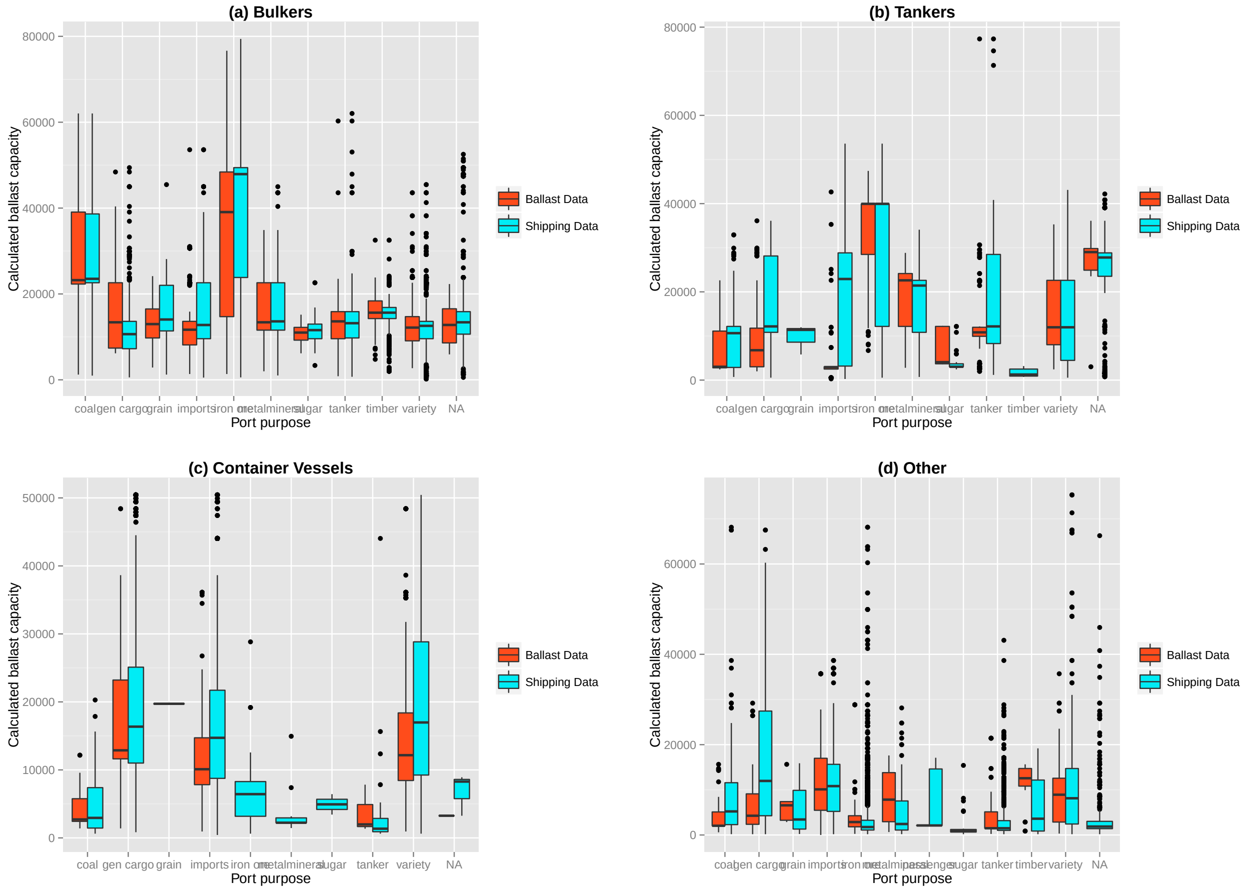

Figure S7: Comparison between the supports of available data in ballast water dataset (1999-2001) and shipping dataset (1999-2012), accross each vessel type and destination port purpose classification.

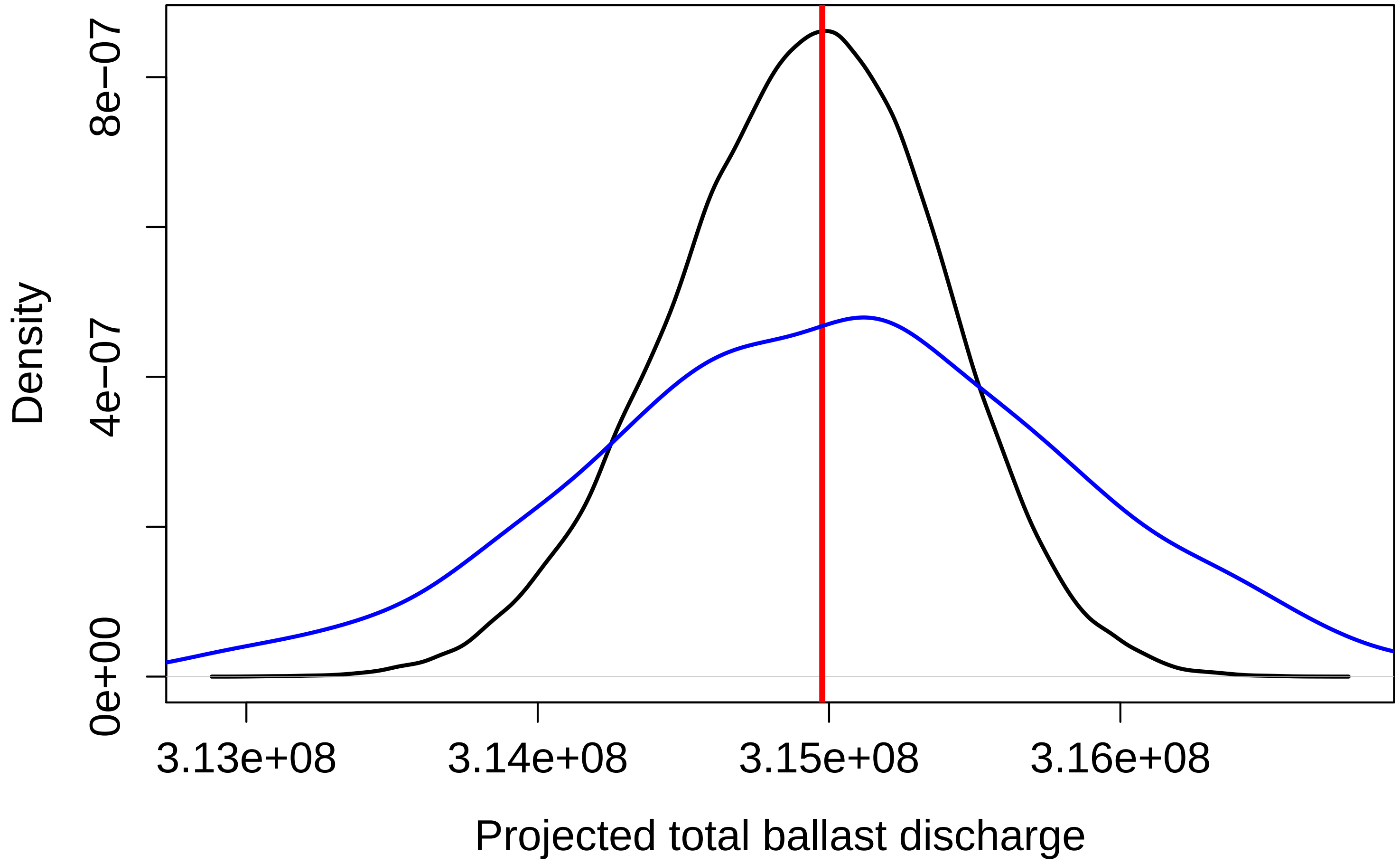

Figure S8: Cross-validated total ballast discharge (1999-2001) for 4-component mixture models (black) and a single-component GLM (blue). Red line indicates the true total ballast volume.

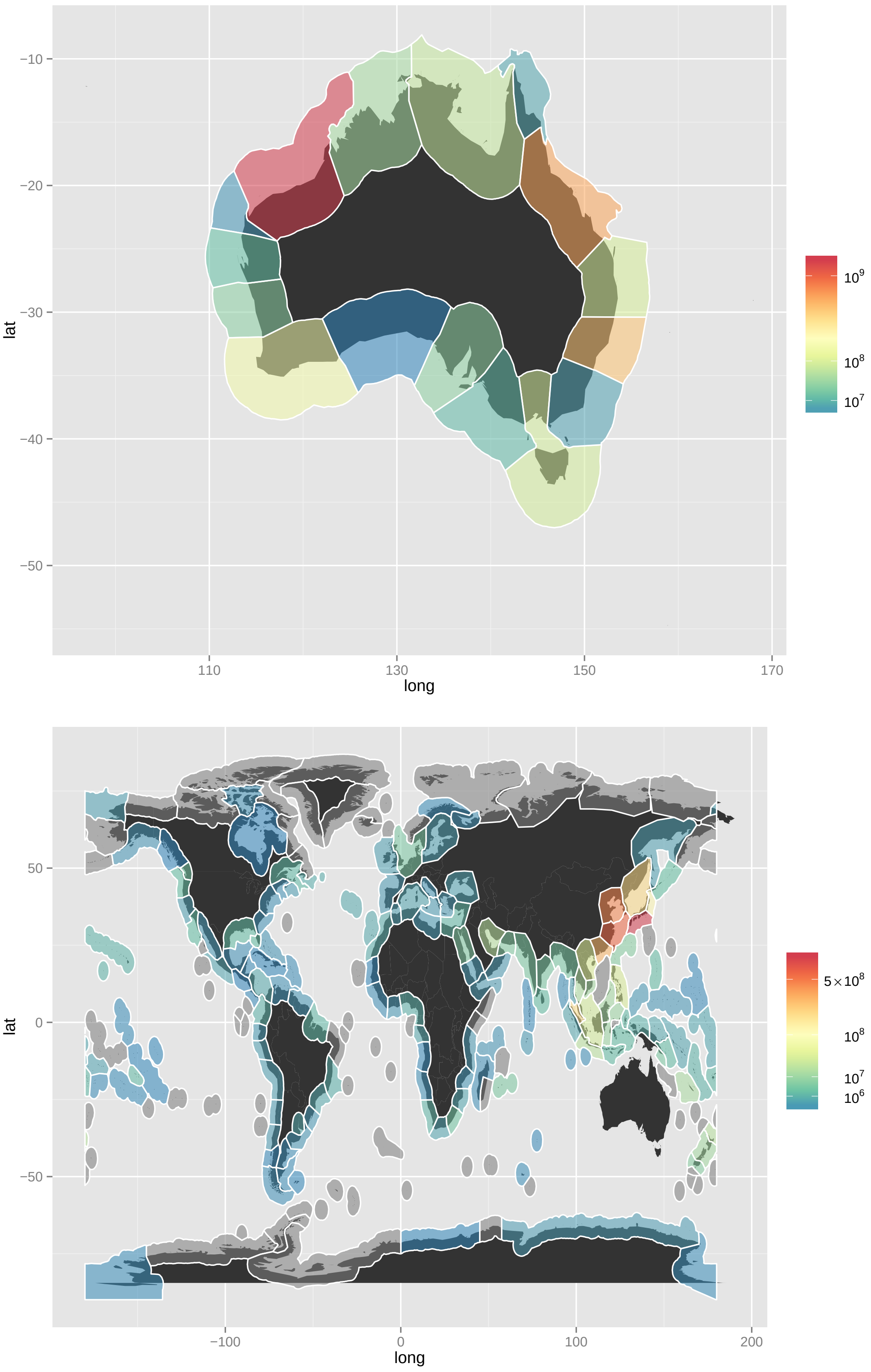

Figure S9: Total ballast water transit volume (m^3), 1999-2012 inclusive, by ecoregion: (a) arrival ecoregion of ballast water, (b) source ecoregion of ballast water. Grey ecoregions had no traffic.

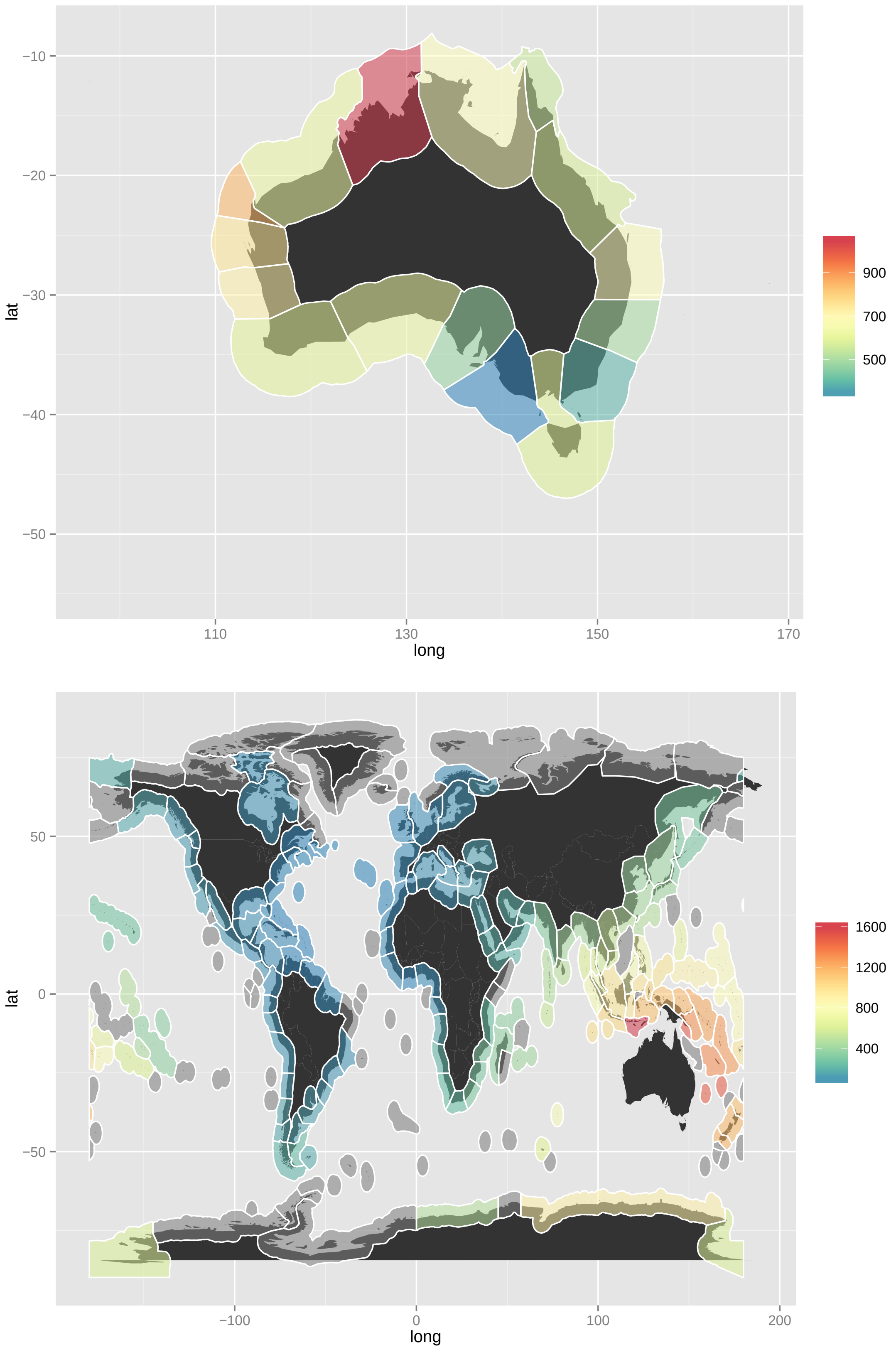

Figure S10: Projected mean density of propagules in ballast water (propagules / m^3), based on vessels (a) arriving to or (b) departing from each ecoregion.

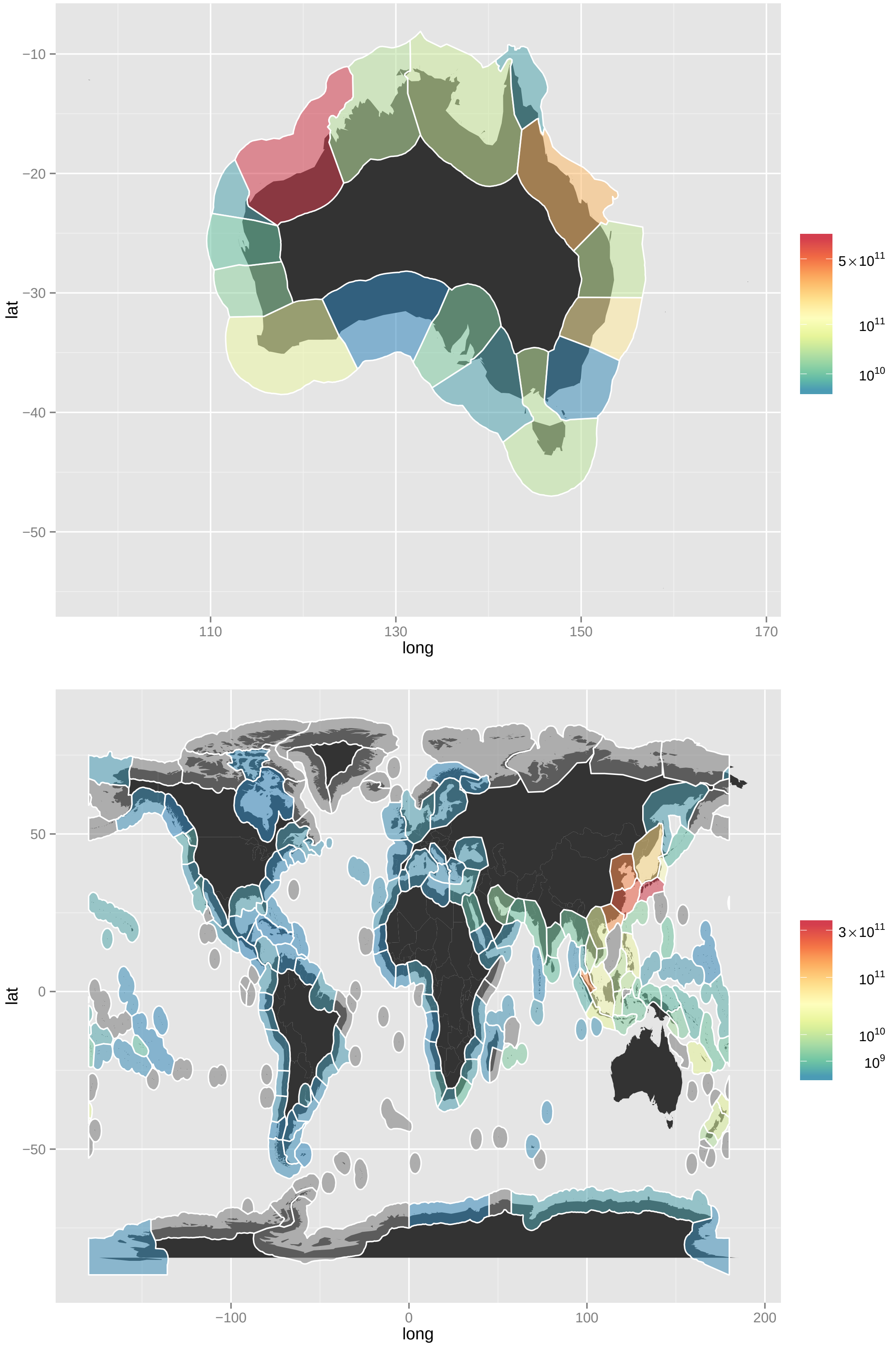

Figure S11: Projected total propagules in ballast across full 1999-2012 shipping dataset.

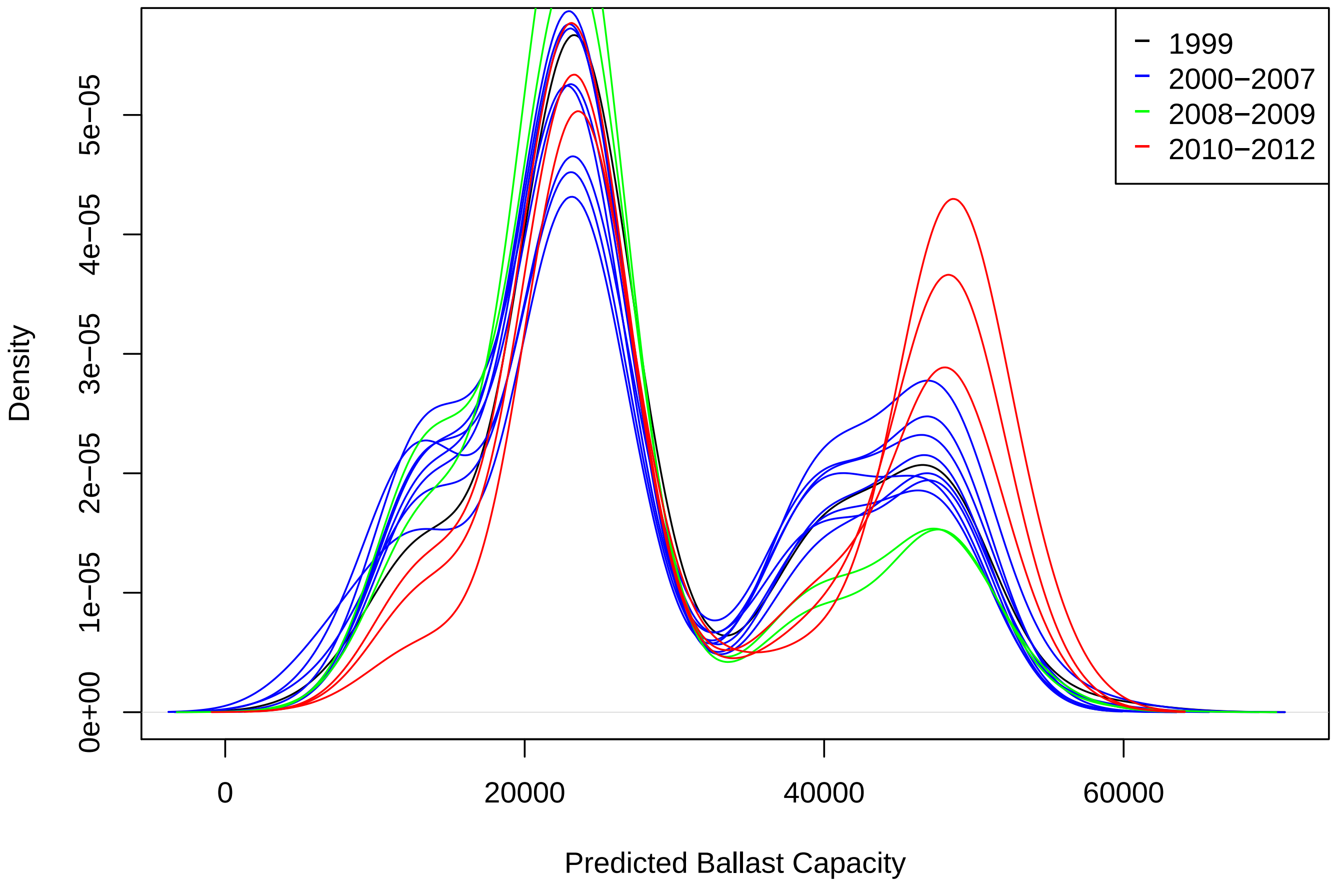

Figure S12: Change in size-distribution of traffic to Dalrymple Bay over time. This port was undergoing expansion during 2008-09.
